# Comparative Genomics Analysis of *Streptococcus* Isolates from the Human Small Intestine Reveals their Adaptation to a Highly Dynamic Ecosystem

**DOI:** 10.1371/journal.pone.0083418

**Published:** 2013-12-30

**Authors:** Bartholomeus Van den Bogert, Jos Boekhorst, Ruth Herrmann, Eddy J. Smid, Erwin G. Zoetendal, Michiel Kleerebezem

**Affiliations:** 1 Top Institute Food and Nutrition (TIFN), Wageningen, The Netherlands; 2 Laboratory of Microbiology, Wageningen University, Wageningen, The Netherlands; 3 Centre for Molecular and Biomolecular Informatics, Radboud University Medical Centre, Nijmegen, The Netherlands; 4 NIZO Food Research B.V., Ede, The Netherlands; 5 Laboratory of Food Microbiology, Wageningen University, Wageningen, The Netherlands; 6 Host-Microbe Interactomics Group, Wageningen University, Wageningen, The Netherlands; University Medical Center Utrecht, The Netherlands

## Abstract

The human small-intestinal microbiota is characterised by relatively large and dynamic *Streptococcus* populations. In this study, genome sequences of small-intestinal streptococci from *S. mitis*, *S. bovis*, and *S. salivarius* species-groups were determined and compared with those from 58 *Streptococcus* strains in public databases. The *Streptococcus* pangenome consists of 12,403 orthologous groups of which 574 are shared among all sequenced streptococci and are defined as the *Streptococcus* core genome. Genome mining of the small-intestinal streptococci focused on functions playing an important role in the interaction of these streptococci in the small-intestinal ecosystem, including natural competence and nutrient-transport and metabolism. Analysis of the small-intestinal *Streptococcus* genomes predicts a high capacity to synthesize amino acids and various vitamins as well as substantial divergence in their carbohydrate transport and metabolic capacities, which is in agreement with observed physiological differences between these *Streptococcus* strains. Gene-specific PCR-strategies enabled evaluation of conservation of *Streptococcus* populations in intestinal samples from different human individuals, revealing that the *S. salivarius* strains were frequently detected in the small-intestine microbiota, supporting the representative value of the genomes provided in this study. Finally, the *Streptococcus* genomes allow prediction of the effect of dietary substances on *Streptococcus* population dynamics in the human small-intestine.

## Introduction

*Streptococcus* is a genus of Gram-positive, low GC-rich species belonging to the lactic acid bacteria (LAB) in the family Streptococcaceae [1]. While several *Streptococcus* species, such as *S. pyogenes* [2] and *S. pneumonia* [3] are recognized as human pathogens, others like *S. salivarius*, *S. mitis*, *S. parasanguinis* are commonly detected as relatively dominant inhabitants in the upper respiratory tract [1], oral cavity [4], [5], throat [6], esophagus [7], stomach [8], and small-intestine [9], [10], [11] of healthy individuals.

Studies carried out by our laboratory focused on elucidating the composition and function of the microbial community in the small-intestine, using ileostoma effluent samples as a representation of the luminal content of the small-intestine [9], [10], [11], [12]. In ileostomy subjects the terminal ileum is connected to an abdominal stoma making this region of the intestinal tract accessible for non-invasive and repetitive sampling of the luminal fraction of the small-intestinal microbiota [9], [10], [11]. The microbial composition in ileostoma effluent resembled the microbiota that resides in the proximal part of the small-intestine from individuals with an intact intestinal tract [11], [13]. Although *Streptococcus* spp. were detected in each of collected ileostoma effluent samples, their relative abundance fluctuated greatly between individuals and even between samples obtained from the same individual [9], [12]. Furthermore, metatranscriptomic analysis of ileostoma effluent identified carbohydrate transport systems, including several phosphotransferase systems (PTS) among the highly represented expressed functions in the small-intestinal streptococci, suggesting that the activity of these bacteria is focused on efficient uptake and fermentation of the available (diet-derived) carbohydrates in the human small-intestine [11]. This is interesting considering that the human small-intestine represents the first region where food components and the intestinal microbiota meet [14], [15].

Based on the above, the *Streptococcus* populations in the small-intestine are predicted to play a prominent role in the metabolic conversion of primary carbohydrates that are present in this ecosystem, and may thereby effectively compete for dietary carbohydrate nutrients with the host mucosa. Therefore, a cultivation approach was employed to obtain representative *Streptococcus* isolates from the small-intestinal ecosystem [12]. Classification of the isolates on the basis of molecular typing methodologies showed that from one ileostoma sample alone 3 different *Streptococcus* species were recovered belonging to the *S. mitis* group, *S. bovis* group, and *S. salivarius* group. Isolates from the *S. salivarius* group could be further divided in 4 genetic lineages (strain level). Although considerable temporal fluctuations of distinguishable genetic lineages were observed when a second sample was collected and investigated one year later, isolates belonging to a single lineage were recovered from both ileostoma effluent samples. Moreover, the *Streptococcus* lineages displayed different carbohydrate conversion and growth patterns [12]. However, the mechanisms underlying the dynamics at the genetic lineage level is unclear. Therefore, specific aspects of the environmental interaction-potential and the metabolic capacity of 6 small-intestinal *Streptococcus* strains were investigated through analysis of their genome sequences in this study. Furthermore, the genomes enabled the comparison with other streptococci from other niches, allowing the identification of genetic targets for strain-specific PCR-based detection in intestinal samples from different individuals.

## Materials and Methods

### Ethics statement

Small intestinal samples used in this study were collected as part of previous projects, results of which are reported elsewhere [9], [12] (Leimena and Van den Bogert, et al., Unpublished data). These studies were approved by the University Hospital Maastricht Ethical Committee, and were conducted in full accordance with the principles of the ‘Declaration of Helsinki’ (52nd WMA General Assembly, Edinburgh, Scotland, October 2000). Subjects were informed about the study orally and in writing and signed a written informed consent before participation.

Fecal samples used in this study were collected as part of a previous project [16], which was approved by the METC of Wageningen University. Subjects were able to understand the written study information and signed an informed consent.

### *Streptococcus* isolates and chromosomal DNA extraction

The isolation of the small-intestinal *Streptococcus* strains and their molecular typing was described previously [12]. In short, isolates were obtained from ileostoma effluent plated on Mitis Salivarius (MS) agar (Becton Dickinson, Breda, the Netherlands) supplemented with Tellurite solution 1% (Becton Dickinson). The streptococcal isolates were classified by DNA fingerprinting into 6 genetic lineages that belonged to the *S. mitis* (1 lineage), *S. bovis* (1 lineage) and *S. salivarius* (4 lineages) species-groups. A randomly picked representative isolate of each lineage was selected for whole genome sequencing.

Genomic DNA of the isolates was extracted from overnight cultures in 10 ml MS medium at 37°C. Cells were pelleted by centrifugation at 7250 g at 4°C for 15 minutes and subsequently frozen at −20°C. Thawed cell-pellets were resuspended in 2 ml THMS (30 mM TRIS-HCl [Sigma, St. Louis, MO, USA; pH = 8.0], 25% (w/v) sucrose [Sigma], and 3 mM MgCl_2_ [Riedel-de Haën, Seelze, Germany]) supplemented with 10 mg/ml lysozyme (Sigma) and 40 µl mutanolysin (Sigma; 5000 U/ml), aliquoted in equal amount into 2 eppendorf tubes, and incubated at 37°C for 30 minutes. After centrifugation at 14.000 g for 5 minutes and discarding the supernatant, cells were resuspended in 100 µl THMS and mixed with 400 µl TES (50 mM TRIS-HCl [Sigma; pH  =  8.0], 20 mM EDTA [Sigma; pH  =  8.0], 50 mM NaCl [Merck], containing 0.5% (v/v) SDS [Ambion, Austin, TX, USA]) and 20 µl Proteinase K (20 mg/ml) followed by incubation at 56°C for 15 minutes. Nucleic acids were subsequently purified by sequential extraction with acid-phenol (Phenol:Water (3.75:1 v/v); pH  =  4.45−5.68; Invitrogen, Carlsbad, CA, USA), acid-phenol:chloroform (1:1), and chloroform (Sigma-Aldrich, Zwijndrecht, Netherlands) using standard procedures as described by Sambrook, et al. [17]. DNA was precipitated from the water-phase by standard ethanol precipitation [17]. After drying, the DNA pellets were dissolved in 50 µl nuclease free water (Promega, Leiden, Netherlands). One µl RNAse A (10 mg/ml; Qiagen GmbH, Hilden, Germany) was added to the solution followed by incubation at 37°C for 30 minutes. Samples were stored at 4°C. DNA quality and concentrations were determined by nanodrop and on a 1.0% (w/v) agarose gel containing 0.4 µg/ml ethidium bromide (Bio-rad).

### Genome sequencing and annotation

DNA from bacterial isolates was sequenced using 454 GS FLX (Roche) technology in combination with titanium chemistry, producing 350−450 bp reads (234,320 ± 86,626 reads per genome), and by using Illumina HiSeq 2000 technology, producing 11,884,010 ± 1,026,060 paired reads of 50 bp per genome from 3 kb mate pair libraries (Table S1; GATC-Biotech, Konstanz, Germany). Pyrosequence reads were assembled using the Celera Assembler v6.1 (http://sourceforge.net/apps/mediawiki/wgs-assembler/index.php?title=Main_Page), and the resulting contigs were subsequently combined with paired-read Illumina sequencing data to generate scaffolds using the SSPACE software v1.1 [18]. Genome pseudo-assemblies were constructed by placing scaffolds in their likely order based on comparisons with the genomes from closely related bacteria: *Streptococcus parasanguinis* ATCC 15912 ([Genbank: NC_015678]; *S. mitis* species-group), *Streptococcus gallolyticus* UCN34 ([Genbank: NC_013798], *S. bovis* species-group), and *Streptococcus salivarius* CCHSS3 ([Genbank: NC_015760]; *S. salivarius* species-group). These comparisons were manually screened for inconsistencies using the Artemis comparison tool [19]. Genomes were annotated using the RAST server [20]. The genes predicted in the genomes of the six small-intestinal isolates were assigned to Cluster of Orthologous groups (COG; [21]) categories, using blastp comparison with the COG database (NCBI, ftp://ftp.ncbi.nih.gov/pub/COG/COG) using an alignment E-value cut-off of 10^−3^.

### Strain identifiers and accession numbers

The Whole Genome Shotgun projects of the human small intestinal *Streptococcus* strains have been deposited at DDBJ/EMBL/GenBank under the following strain identifiers (and accession): the *S. parasanguinis* strain from the *S. mitis* species-group: HSISM1 (ASKI00000000), the *S. equinus* from the *S. bovis* species-group: HSISB1 (ASKA00000000), *S. salivarius* strain 1: HSISS1 (ASKB00000000), *S. salivarius* strain 2: HSISS2 (ASKC00000000), *S. salivarius* strain 3: HSISS3 (ASKH00000000), and *S. salivarius* strain 4: HSISS4 (ASKD00000000). The version described in this paper is version XXXX01000000.

### Genome orthology

Orthology relationships were identified by comparing all predicted gene products from all 6 small-intestinal *Streptococcus* genomes with the genes predicted to be encoded by the 58 other *Streptococcus* genomes (See Table S2 for accession numbers) that were available within the NCBI database on February 22^nd^, 2012 using OrthoMCL v2.0.2 with default parameters. Genome metadata (e.g. isolation site) from the *Streptococcus* genomes was retrieved from the Genome OnLine database (GOLD; http://genomesonline.org) on February 27^th^, 2012 (Table S2).

### *Streptococcus* phylogenetic tree reconstruction

Multiple protein sequence alignments of the 450 orthologous groups with exactly one member in each *Streptococcus* genome were generated using MUSCLE [22]. The variable positions were concatenated into a single alignment (length 5605 residues) and a maximum-likelihood phylogenetic tree was generated using PhyML [23]. The phylogenetic tree was visualized using the TREEVIEW program [24].

### Genome mining and metabolic mapping

Bacterial genomes were mined for systems involved in responses to external stimuli, focusing on bacteriocins, identified using BAGEL2 employing no re-annotation [25], and two-component systems (TCS) consisting of sensor histidine kinase (HK) and response regulator (RR) pairs [26].

Moreover, genomes were screened for gene clusters involved in regulation of natural competence: comCDE, present in the *S. mitis* group species, or comRS in *S. bovis* and *S. salivarius* streptococci [27].

Protein sequences for genes annotated as transposases were assigned to Insertion Sequences (IS; See [28] for a review) families using blastp comparison with the ISfinder database [29]. IS families were assigned based on the best hit. Multiple alignment of the protein sequences of transposases were performed using ClustalX2 [30]. Small interspersed repeats (e.g. BOX elements [31], Repeat Unit of Pneumococcus (RUP) [32], [33], and *Streptococcus pneumoniae* Rho-Independent Terminator-like Element (SPRITE) [34]) were identified with HMMER2 [35] using the Hidden Markov Models (HMMs) generated for *S. pneumoniae* and *S. suis* by Croucher, et al. [34].

Genomes were further screened for sugar transport systems including constituents of the bacterial phosphotransferase system and ABC transporters. Metabolic and amino acid biosynthesis pathways were constructed for the newly sequenced genomes by mapping EC numbers from the genome annotations onto the Kyoto Encyclopedia of Genes and Genomes (KEGG) metabolic pathways [36]. Pathways from individual KEGG maps that were represented in at least one of the *Streptococcus* genomes were included in combined metabolic visualizations for sugar metabolism and amino acid biosynthesis that were manually constructed. In cases where genes of key enzymes in specific pathways of interest were apparently absent from the genome-based predictions, a further effort was made to identify homologous gene candidates by dedicated BLAST searches [37].

### Unique gene identification and PCR detection

Each of the newly sequenced genomes was screened for ‘unique’ genes that were not present in other small-intestinal *Streptococcus* genomes or other genomes in the NCBI database. Single copy unique genes with a sequence length of at least 750 nt were used for primer design employing the Primer-BLAST tool (http://www.ncbi.nlm.nih.gov/tools/primer-blast/), which uses the Primer3 program [38]. Default parameters were used, except for the following changes: PCR product size: 150 to 300 bp; maximum primer size: 23 nt; minimum GC content: 40%; maximum poly-X (mononucleotide repeats): 3; maximum self-complementarity: 3.

Primer specificity was checked by submitting each primer to Primer-BLAST using genomes, “Genomes (chromosomes from all organisms)” from all Bacteria, as a reference database. An in-house perl script was used to determine if the primers designed had exact matches in small-intestinal *Streptococcus* genomes other than the intended *Streptococcus* strain target. This revealed that primers developed for *S. salivarius* lineage 4 were not exclusively specific for the intended target strain, but were predicted to be cross reactive with *S. salivarius* lineage 1. By decreasing the minimal gene sequence length to 500 nt, primers were developed that were specific for *S. salivarius* lineage 4.

After the recent addition of novel *Streptococcus* genomes to the DNA databases a reassessment of primer specificity showed that primers targeted to the *S. salivarius* strains of lineage 1 and 4 (HSISS1 and HSISS4) have predicted matches in the genome of *S. salivarius* JIM8777. However, the predicted PCR-products are exceptionally large (e.g. >4000 bp) and/or the primers show between 1 and 5 mismatches with the *S. salivarius* JIM8777 target. These features make it highly unlikely that a PCR-amplicon is formed using employed PCR conditions. The primers targeted to HSISS4 are predicted to form a PCR-product for which the primers only show a single mismatch with *S. salivarius* JIM8777. Therefore, the primers targeted to the *S. salivarius* strains of lineage 1 and 4 (HSISS1 and HSISS4) may not be exclusively specific to their intended targets, but can be used to detect streptococci belonging to the *S. salivarius* species group that carry the target functional gene.

Primers that passed each screening step, were specific for their target strain, and had a minimal tendency to form secondary structures, including hairpin loops, heterodimers, and homodimers (analysed by the IDTDNA Oligoanalyzer 3.1; Integrated DNA Technologies) were ordered (Biolegio BV, Nijmegen, Netherlands) and tested for their application in strain specific PCR detection assays (see below; Table S3).

All PCRs were performed on a C1000™ Thermal Cycler (Bio-rad) with a CFX96 optic module (Bio-rad) employing CFX Manager 2.1 (Bio-rad) software for analysis. Reactions were carried out in Hard-Shell semi skirted clear 96 well plates (Bio Rad) sealed with Microseal B film (Bio Rad) in 25 µl volumes using IQ SYBR green supermix (Bio-Rad) according to the manufacturer’s instructions with 200 nM of forward and reverse primer and either 5 µl gDNA (10−20 ng/µl) or glycerol stock of the strain as a template source.

The optimal annealing temperature (60°C) for each primer pair was determined by an 8-degree temperature (53°C to 64°C) gradient PCR using gDNA from target strains as template (data not shown).

The PCR program started with a denaturation step at 95°C for 5 minutes, followed by 40 cycles consisting of denaturation at 95°C for 15 s, annealing for 60°C for 30 s and elongation at 72°C for 20 s with data collection, and a final elongation step at 72°C for 10 minutes. Ct values above 35 were considered negative. Melting curve analysis was carried out by incrementally increasing the temperature from 55°C to 95°C at 30 s per 0.5°C with continuous fluorescence collection. Control PCRs were performed alongside each separate amplification without addition of template and consistently yielded no product.

### Small-intestinal and fecal sample collection

In total, 30 ileostoma effluent samples were collected in the morning or afternoon (at least 3 h apart) on separate days (at least two days apart) from 6 ileostomy subjects (4 male and 2 female; aged 55 to 79; A-F), as part of previous projects, results of which are reported elsewhere [9], [12] (Leimena and Van den Bogert, et al., Unpublished data). Small-intestinal fluid samples were obtained from 3 healthy individuals (3 males; 24 ± 4.5 years; G-I) and included a jejunal sample and an ileum sample from subject H and a single ileum sample from subjects G and I [11]. Fecal samples were collected from 10 individuals (4 male and 6 female; aged 19 to 33; J-S) as part of a previous project [16].

DNA was extracted using the Repeated Bead Beating method described in [39] or using a method adapted from Zoetendal, et al. [12], [40], [41], depending on the study they originated from, and was used to screen for the unique targets of the *Streptococcus* genetic lineages.

## Results

### General features of small-intestinal streptococcal genome sequences

**Figure 1:**
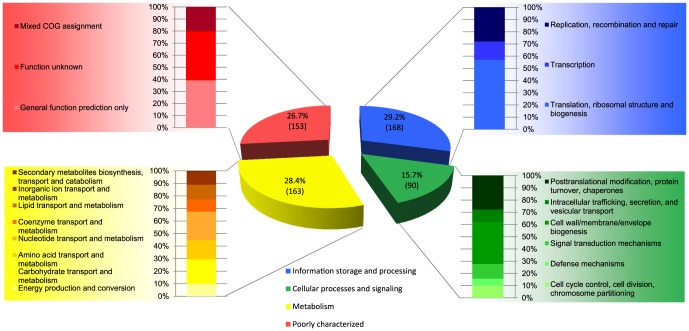
Distribution of COG classifications for 574 OG in the core *Streptococcus* genome.

The entire genome set analysed in this study consisted of 64 genomes, encompassing 20 *Streptococcus* species. Six draft genome sequences were obtained from strains originating from the small-intestine, which were determined in this study and ranged in genome size from 1.9 Mbp (*Streptococcus* strain from the *bovis* species-group) to 2.4 Mbp (*S. salivarius* lineage 3; See table S1 for genome statistics). The full complement of genes (pangenome) of the *Streptococcus* genome set consisted of 12,403 orthologous groups (OG), of which 4,232 OG were represented in the genomes of at least one of the six small-intestinal *Streptococcus* strains. The size of the *Streptococcus* pangenome estimated here is somewhat larger as has been suggested in previous studies [42], [43]. However, these studies based their pangenome estimates on a smaller genome set comprising fewer species. Furthermore, the *Streptococcus* pangenome defined here does not seem to be exceptionally high compared to, for example, the *Lactobacillus* pangenome estimated to consist of over 13,000 protein-encoding genes [44] or gene families [43]. Further analysis revealed that all 64 *Streptococcus* genomes shared 574 orthologous groups (OG), defining the core *Streptococcus* genome. All OG belonging to the core *Streptococcus* genome could be classified to a COG, although 26% of these OGs was assigned to poorly characterized COG categories (Figure 1). Most OG in the core *Streptococcus* genome were predicted to be involved in information storage and processing (29.2%), with most genes belonging to typically conserved functions such as ‘Translation, ribosomal structure and biogenesis’ and ‘Replication, recombination and repair’. Metabolic functions accounted for 28.4% of the core *Streptococcus* OG, followed by 15.7% of OG that were involved in cellular processes and signalling. Most OG belonging to ‘metabolism’ were assigned to functions in transport and metabolism of nucleotides and carbohydrates.

**Figure 2:**
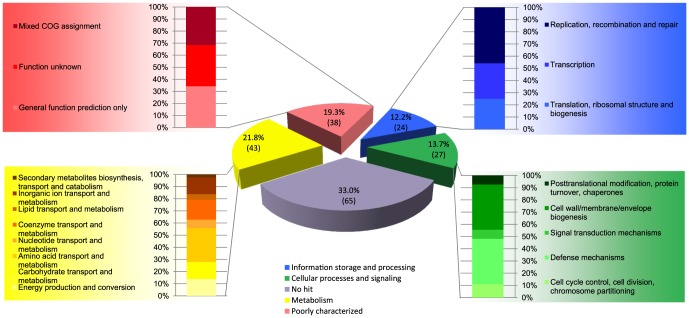
Distribution of COG classifications for 197 OG represented in 2−6 small-intestinal genomes.

Most streptococcal genome sequences from the public databases that were included in the analysis here, were derived from clinical bacterial isolates cultivated from different human body sites (Table S2). Due to frequent obscurity concerning the source of isolation it is far from trivial to identify niche-specific OG. Nonetheless, 197 OG, mostly belonging to ‘metabolism’ (Table S4), were found to be present in at least two of the small-intestinal *Streptococcus* genomes, but not in any of the genomes from the public databases. An assessment of the orthology relationships between the *Streptococcus* strains analysed here as well as the recently made available genome of *Streptococcus* sp. HPH0090 (accession: NZ_ATCD00000000), a strain isolated from a biopsy of ileal-anal pouch mucosa as part of the Human Microbiome Project (http://www.hmpdacc.org) [45], revealed that only a fraction of the OG were shared by three or more small-intestinal *Streptococcus* genomes (e.g. 10 genes of HPH0090 belonged to the 197 OG). This suggests that there is considerable variation in the number of streptococcal genes with functions that may contribute to the lifestyle in the small-intestine ecosystem. Notably, a considerable fraction of the 197 OG (65; 33.0%) could not be assigned to a COG, and were predominantly annotated as hypothetical proteins (44; Figure 2). This suggests that for a substantial amount of OG that in this analysis were exclusively encountered in the streptococcal genomes from the small-intestine, the function needs to be further elucidated (Figure 2).

### Phylogenetic analysis of *Streptococcus* genomes

**Figure 3:**
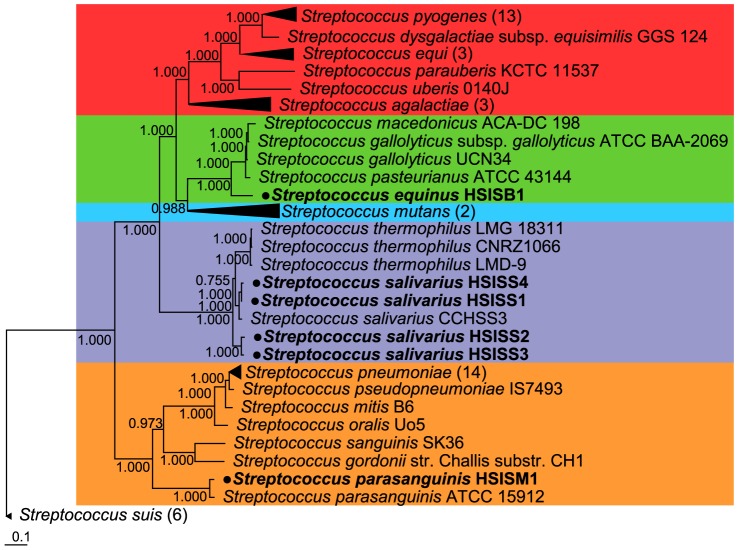
*Streptococcus* phylogenetic tree. Unrooted maximum-likelihood phylogenetic tree based on multiple protein sequence alignments (length 5605 residues) of the 450 orthologous groups with exactly one member in 64 *Streptococcus* genomes. Genomes belonging to the Pyogenic group are highlighted in red, *S. bovis* species-group in green, *S. mutans* species-group in blue, *S. salivarius* species-group in purple, and *S. mitis* species-group in orange. Small-intestinal *Streptococcus* strains are highlighted and bulleted. Visualisation of the nodes (i.e. multiple leaves) of the *S. pyogenes*, *S. equi*, *S. agalactiae*, *S. mutans*, *S. pneumoniae*, and *S. suis* genomes were collapsed into triangles. Values in the tree are approximate likelihood ratio test (aLRT scores (SH-like) as calculated by PhyML).

The subset of genes of the core *Streptococcus* genome (450) that were present in single copy in each genome was used to construct a phylogenetic tree (Figure 3). This core-genome-based phylogeny revealed a division of 5 distinct clusters of *Streptococcus* strains that belong to the *Streptococcus* species-groups: Pyogenic (e.g. *S. pyogenes*), *S. bovis*, *S. mutans, S. salivarius*, and *S. mitis* groups. *S. suis* genomes could not be assigned to one of these *Streptococcus* species-groups [1], but represented an additional and separate phylogenetic branch (Figure 3). Notably, 5−140 orthologous groups were exclusively present in all genomes belonging to one of the clusters (cluster-specific genes) and might be used as cluster-markers for molecular detection and quantification (Table S5). The small-intestinal *Streptococcus* genomes clustered within the *S. mitis*, *S. bovis*, and *S. salivarius* groups, corroborating previous classifications based on MALDI-TOF MS analysis and 16S rRNA gene analysis [12], which showed that the strain belonging to the *S. mitis* species-group showed highest similarity to *S. parasanguinis* (> 99%), the strain from *S. bovis* species-group showed highest similarity to *S. equinus* (> 98.5%) and *S. lutetiensis* (> 99.7%;), and the strains belonging to the *S. salivarius* species-group showed highest similarity to *S. salivarius* subsp. *salivarius* (> 98.7%) and *S. vestibularis* (> 99.3%). To specify the classification of the isolate belonging to the *S. bovis* species-group a phylogenetic analysis of the SodA encoding gene [46] was performed, revealing that the small-intestinal strain clustered together with strains from the species *S. equinus* (data not shown). To improve species classifications of the salivarius group streptococci, we focused on the genetic organization of the region encoding the transketolase gene, that is known to differ between *S. salivarius* and *S. vestibularis* [47]. In the genomes of all small-intestinal strains belonging to the *S. salivarius* species-group the transketolase gene is situated in a region flanked by genes encoding triose phosphate isomerase and thymidylate kinase, which is typical for *S. salivarius* species ([47]; data not shown).

The distance between the strains from *S. salivarius* lineage 1 and 4 was relatively small (Figure 3), which is in agreement with the similarity of fermentation and growth [12] as well as the immunostimulatory (Van den Bogert and Meijerink, et al., Unpublished data) profiles that were previously determined for these strains. To further assess the similarity between *S. salivarius* lineage 1 and 4, the number of shared genes was determined. This revealed that both lineages shared 1730 OG, which is high compared to the number of shared genes between the two other *S. salivarius* lineages (Table S6). Nonetheless, *S. salivarius* lineage 1 and 4 were still predicted to have 128 and 237 strain-specific OG, respectively. The set of 128 lineage 1 specific OGs were manually inspected for potential sequencing and/or gene-calling artifacts (See table S7). These genome sequence analyses confirmed that the representative isolates of *S. salivarius* lineages 1 and 4 are closely related, and confirmed and extended our previous observations based on AFLP and Rep-PCR fingerprinting [12].

### Genomic mining to decipher environmental interaction potential

To obtain an impression how the analysed *Streptococcus* strains may react to external stimuli, we mined their genomes for the canonical TCS regulatory modules, consisting of HK and RR, that are known to play a prominent role in bacterial interaction with their environment [26]. The strains appeared to encode 12−18 HK/RR) pairs, which are predicted to respond to a wide variety of environmental responses (Table S8). TCS annotated as CiaRH, ComDE, VraSR, and CsrSR were identified in all strains.

The CiaRH system responds to environmental Ca^2+^ [48] and has been shown to be important for intracellular survival of group B *Streptococcus* [49]. It has been shown that this system is involved in regulation of numerous functions in *S. pneumoniae*, including those associated with natural competence, which is a driver of evolution [27], [50]. Analogously, the *comCDE* encoded TCS (ComDE), present in the *S. mitis* group species [27], has been shown to be the central regulatory module in the control of natural competence, involving a *comC* encoded extracellular competence stimulatory peptide (CSP) as its autoregulatory environmental cue [51]. The small-intestinal *S. parasanguinis* strain appeared to encode two candidate *comDE* TCS, but a putative CSP encoding *comC* gene upstream of *comDE* could not be identified. The *S. equinus* and *S. salivarius* strains from the small-intestine were found to encode a distinct competence regulatory module consisting of a transcriptional regulator and a putative oligopeptide pheromone, that share similarity with ComR and ComS [27],[52] and are genetically linked to conserved *comX* promoter structures [50]. The oligopeptide predicted for the *S. equinus* strain (MKVFSILLTGWWLG) contains the conserved double-tryptophan (WW) motif, which is a conserved feature of ComS from bovis streptococci [50]. The oligopeptides predicted for the strains from *S. salivarius* lineage 1 and 4 are identical (MKKLKLFTLFSLLITILPYFAGCL) and resemble that of *S. salivarius* SK126 [50], [52], but have a single amino acid substitution (“T” in *S. salivarius* SK126 to “A” in *S. salivarius* lineage 1 and 4) in the predicted 7 amino acid long C-terminal peptide that is likely to prevent inter-strain crosstalk. Moreover, the lineage 1 representing strain appears to contain a frameshift in the region encoding the N-terminal end of the oligopeptide. The oligopeptides predicted for the salivarius strains from lineage 2 and 3 are also identical, but are distinct (MKNLRKFLVLLIAAAPFFIIYY) from the sequence presented above. It is likely that competence could be induced in these strains via extracellular addition of the unmodified small peptides, especially since all genomes presented here appear to encode a complete competence regulon including genes encoding a competence specific sigma factor ComX, and late competence complexes (e.g. *comEA*/*C*, and *comGA*/*B*/*C*/*D*/E/F/*G*), which are involved in DNA uptake and DNA processing (see [27] for review).

To investigate if there is any evidence of horizontal gene transfer among the small-intestinal streptococci their genomes were mined for mobile elements, including IS, RUP, BOX and SPRITE. This revealed that the strains appeared to encode 1 (*S. equinus* HSISB1) to 22 (*S. salivarius* HSISS3) transposases that belonged to 7 IS families (IS3, IS30, IS1182, IS200/IS605, IS110, ISL3, and IS256). While *S. equinus* HSISB1 encoded a single transposase belonging to the IS 30 family, *S. parasanguinis* HSISM1 encoded 10 transposases that belonged the IS3 family. The latter could be further divided into 3 groups based on alignment of the protein sequences. The transposases encoded by the *S. salivarius* strains belonged to different families (IS3, ISL3, IS26, IS30, IS110, IS200/IS605, and IS1182). Interestingly, transposases from the IS3, ISL3, and IS256 families encoded in the genomes of the *S. salivarius* strains aligned perfectly suggesting that there is genetic exchange between the streptococci in the small-intestine (Figure S1). Though the HMMs produced by Croucher, et al. [34] are based on alignment of repeat sequences from *S. pneumoniae* and *S. suis*, their application to the small-intestinal *Streptococcus* genomes found 14 BOX elements, present in all genomes except in that of *S. equinus*, and 3 SPRITE elements, exclusively present in the *S. parasanguinis* genome, indicating that horizontal gene transfer could occur between small-intestinal streptococci (Table S9).

The genomes of all the small-intestinal *Streptococcus* strains described here, appeared to encode a TCS that resembles the NisK-NisR and/or SpaK-SpaR TCS modules involved in quorum-sensing controlled autoregulation of nisin and subtilin biosynthesis in *Lactococcus lactis* and *Bacillus subtilis*, respectively (for a review see [53]). Both nisin and subtilin are antimicrobial peptides (bacteriocins) that contain extensive post-translational modification and belong to the class of the lantibiotics (for a review see [54]) and their biosynthesis depends on multi-gene clusters encoding modification, export, immunity, and the mentioned TCS functions [53]. To investigate whether the identified streptococcal homologues of these lantibiotic TCS may be involved in regulation of lantibiotic production by these strains, the genetic context of the TCS encoding genes was investigated. However, this analysis failed to identify additional genes that were predicted to be involved in lantibiotic biosynthesis in these organisms. To perform a genome wide analysis of the capacity to produce antimicrobial peptides, we employed the BAGEL2 software module [25] that identified at least one putative bacteriocin encoding gene in the genomes of the *S. equinus* and *S. salivarius* strains. All candidate genes belonged to the non-lanthionine-containing bacteriocins of the pediocin-like (class IIA) and/or miscellaneous (class IID) class according to the scheme proposed by Cotter, et al. [55] (Table S10). This analysis indicates that despite their resemblance to NisRK-like TCS modules, these TCS systems are not involved in regulation of genetically linked or distantly located lantibiotic encoding gene clusters, and are thus most likely involved in regulation of other functions.

All *Streptococcus* strains analysed here appeared to encode the CsrSR system, although *S. parasanguinis* appeared to lack a HK paired to the RR similar to CsrR. The CsrSR TCS module is known to play a major role in regulating the virulence of group A and B streptococci [56], [57]. Group A streptococcal CsrSR regulates the expression of virulence factors (e.g. pyrogenic exotoxin A, DNase, streptolysin O, streptokinase, and hyaluronic acid capsule synthesis) depending on environmental Mg^2+^, as well as human antimicrobial peptide LL-37 concentrations [57]. The CsrSR TCS in group B streptococci is known to repress the expression of certain genes (e.g. coding for β-haemolysin and secreted adhesins) while it stimulates expression of other genes (e.g. the *cps* operon coding for capsular polysaccharide [56]). All newly sequenced *Streptococcus* genomes were predicted to encode genes with similarity to hemolysin III. However, only the *S. parasanguinis* and *S. equinus* strains displayed partial (α) hemolysis and none displayed complete (β) hemolysis of blood cells when grown on blood agar (data not shown). The streptococcal genomes were predicted to encode capsular polysaccharide biosynthesis and appeared to encode a gene similar to exfoliative exotoxin B. The strains representing *S. salivarius* lineage 1 and 4 also contained a gene with homology to the C5a peptidase precursor. The latter enzyme inactivates C5a, a chemotactic attractant of phagocytes to infection sites, and promotes streptococcal invasion [58], [59]. Although the strains described here are not known to be virulent, they appear to encode at least remnants of the virulence genes known in related streptococci, which may be regulated by the conserved CsrSR TCS module, analogous to what is observed for group A and B streptococci. Remnants of virulence related genes were also encountered in the genomes of strains of *S. themophilus* [60], suggesting that these benign streptococci share specific functions with their known pathogenic relatives.

The VraSR TCS that appeared to be encoded by all streptococcal genomes reported here, has been extensively studied in *Staphylococcus aureus* where it belongs to the cell-wall-stress stimulon that is involved in maintenance of cell wall integrity under stress conditions [61]. In *S. aureus* VraSR plays an important role in regulation of resistance to antibiotics that target the bacterial cell wall biosynthesis pathway. Whether the VraSR homologues in the small-intestinal streptococci play a similar role in cell-wall stress and possible antibiotic resistance control remains to be established.

### Amino acid and vitamin requirements

The predicted enzyme functions of the newly sequenced *Streptococcus* genomes were mapped onto KEGG pathways to assess their predicted potential for amino acid biosynthesis. Each of the genomes was predicted to encode the necessary enzymes for the biosynthesis of at least 18 amino acids (Table 1; Figure S2). However, none of the strains found to encode the enzymes required to synthesize lysine. Moreover, the biosynthesis of histidine from the pentose phosphate pathway intermediate phosphoribosyl pyrophosphate (PRPP) appears to be incomplete in the genomes of the *S. salivarius* strains representing lineage 2 and 3, as well as the *S. parasanguinis* strain. Alanine biosynthesis appeared to depend on distinct enzymatic conversion of pyruvate to alanine, involving alanine transaminase dehydrogenase (EC 2.6.1.2) in the *S. equinus* strain and the *S. salivarius* strain representing lineage 3, while involving an alanine dehydrogenase (EC 1.4.1.1) in all other strains (Figure S2).

**Table 1:** Predicted amino acid requirements for growth of newly sequenced *Streptococcus* strains.

Amino acid	*S. parasanguinis*	*S. equinus*	*S. salivarius*			
			1	2	3	4
Arginine	+	+	+	+	+	+
Histidine	−	+	+	−	−	+
Lysine	−	−	−	−	−	−
Aspartate	+	+	+	+	+	+
Glutamate	+	+	+	+	+	+
Serine	+	+	+	+	+	+
Threonine	+	+	+	+	+	+
Asparagine	+	+	+	+	+	+
Glutamine	+	+	+	+	+	+
Cysteine	+	+	+	+	+	+
Glycine	+	+	+	+	+	+
Proline	+	+	+	+	+	+
Alanine	+	+	+	+	+	+
Valine	+	+	+	+	+	+
Isoleucine	+	+	+	+	+	+
Leucine	+	+	+	+	+	+
Methionine	+	+	+	+	+	+
Phenylalanine	+	+	+	+	+	+
Tyrosine	+	+	+	+	+	+
Tryptophan	+	+	+	+	+	+

−: required for growth; +: not required for growth.

Although the small-intestinal streptococci encode the capacity for synthesis of the majority of the amino acids, they also were predicted to encode the oligopeptide import system, *oppABCDF* [62], but lacked a gene resembling an extracellular protease function (e.g. PrtP; [63]). These findings may reflect the adaptation to the peptide and exogenous protease-rich environment that is probably encountered in the human small-intestine.

Next we investigated the predicted capacity to produce B-vitamins, which is known to be variable among streptococci [64]. Genome analyses indicate that all small-intestine derived streptococci presented here encode the capacity to produce folate from phenylalanine. All strains, except *S. parasanguinis*, also appeared to encode a complete pyridoxal-5-phosphate (B6) biosynthetic pathway. In addition, *S. equinus* was predicted to also encode the capacity to synthesize riboflavin (B2), nicotinate (B3), and pantothenate (B5), which appear to be lacking in *S. parasanguinis* and *S. salivarius*. None of the strains is predicted to encode thiamine (B1), biotin (B8) and cobalamin (B12) biosynthesis pathways.

### Primary carbon metabolism and pyruvate dissipation

As streptococci belong to the facultative heterofermentative LAB and generate energy through homolactic and mixed acid fermentation [65], we screened the genomes of the small-intestinal streptococci for genes involved in glycolysis and the pentose phosphate pathway. All strains encoded the required enzymes for glycolytic conversion of glucose to pyruvate (Table S11). Notably, only the *S. parasanguinis* strain appeared to encode a complete and intact pentose phosphate pathway. However, the *S. equinus* and *S. salivarius* strains, appeared to code for a transketolase (EC 2.2.1.1) that interconnects the glycolysis and the pentose phosphate pathway, enabling the synthesis of the precursor required in *de novo* purine and pyrimidine synthesis, phosphoribosyl pyrophosphate (PRPP; Figure S3). In addition, the *S. equinus* strain, codes for a putative xylulose-5-phosphate phosphoketolase (EC 4.1.2.9; Table S11 and figure S3), suggesting that this strain can ferment pentoses (e.g. arabinose [12]; see below) that enter the pentose phosphate pathway as xylulose-5-phosphate. As expected, genomic analyses showed that none of the small-intestinal streptococci code for a complete tricarboxylic acid (TCA) cycle, albeit that several enzymes (e.g. EC 1.3.99.1, Fumarate reductase) from this pathway are predicted in the genome annotations. The presence of fumarate reductase in the genomes may indicate that these streptococci possess a rudimentary electron transport chain, similar to what is observed for other LAB, including *L. plantarum* WCFS1 [66].

As expected, all the small-intestinal *Streptococcus* genomes have the necessary enzymes to convert pyruvate to L-lactate (lactate dehydrogenase [EC 1.1.1.27]). Although the genomes appeared to lack the genes to produce a complete pyruvate dehydrogenase complex, they do encode the necessary enzymes for mixed acid fermentation via the formate lyase (EC 2.3.1.54), phosphate acetyltransferase (EC 2.3.1.8), and acetate kinase (EC 2.7.2.1) pathway. In addition, the genomes also encompass acetaldehyde dehydrogenase (EC 1.2.1.10) and alcohol dehydrogenase (EC 1.1.1.1) encoding genes, implying their capacity to produce ethanol (Figure S3). Finally, all streptococci appeared to encode both acetolactate synthase (EC 2.2.1.6) and acetolactate decarboxylase (EC 4.1.1.5) that could catalyze the conversion of pyruvate to acetoin.

### Sugar metabolism

**Figure 4:**
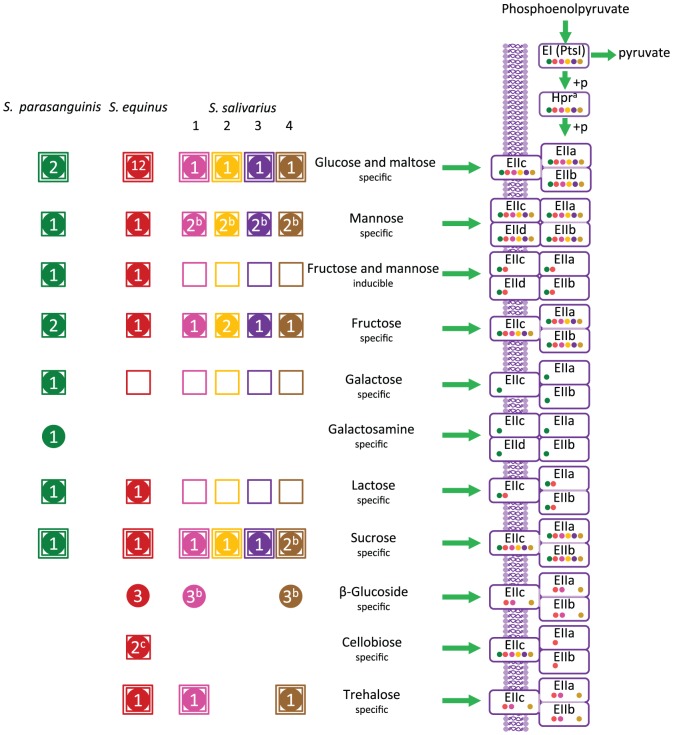
Overview of PTS in six sequenced genomes from small-intestinal *Streptococcus* strains. Dots indicate for which genome the corresponding PTS (component) was encoded. Numbers in dots represent the number of times a complete PTS complex was encoded in the genome. Squared dots indicate which isolates were able to ferment the corresponding substrate, double squared dots indicate which isolates were able to ferment and grow on the corresponding substrate [12]. Components of PTS that are encoded by the same orthologous group are indicated with faded purple lines ^a^: *S. parasanguinis* and *S. salivarius* lineage 1 carried an additional phosphocarrier protein (Hpr), each belonging to different orthologous compared to the Hpr protein that belonged to the same OG that was shared between all *Streptococcus* ^b^: complete PTS were encoded by genes belonging to different OG ^c^: Component EIIa and EIIb from 2 complete cellobiose specific PTS were encoded by genes belonging to the same OG while component EIIc of the two systems belonged to different OG.

*Streptococcus* spp. have been proposed to contribute to microbial uptake and fermentation of the simple dietary carbohydrates in the small-intestine [11]. Therefore, we especially focused our genome annotation efforts on carbohydrate transport functions and metabolism that can be used as fuel for the downstream energy-generating pathways (e.g. glycolysis and pentose phosphate pathway). All small-intestinal strains encode the general cytoplasmic enzyme I (EI) and phosphor-carrier protein (HPr; Figure 4) involved in phospho-donation to several PTS transport systems. In total, 11 distinct PTS transporter functions were found to be encoded by the small-intestinal *Streptococcus* genomes. Those with predicted specificities for glucose/maltose, mannose, fructose, sucrose, β-glucosides, and trehalose were redundantly present in some of the genomes (Figure 4).

All sequenced genomes were predicted to encode complete enzyme II (EII) PTS complexes, consisting of IIa, IIb, and IIc (as well as IId in some cases) components, involved in import of glucose/maltose, mannose, fructose, and sucrose (Figure 4).

Complete PTS transport systems with predicted specificity for cellobiose, β-glucosides, and trehalose appeared to be encoded in the *S. equinus* genome, while the PTS-mediated import capacity for the latter two substrates was also predicted for the lineage 1 and 4 representing *S. salivarius* isolates. Next to these complete PTS EII complexes, all genomes also appeared to encode orphan PTS EIIc transport component(s), which lack the accompanying EIIb and EIIa encoding genes, and in all cases were predicted to have a cellobiose substrate-specificity. This is a feature that has been recognized in many other bacterial genomes, and has been proposed to play a role in environmental signalling [66], [67], [68], [69]. The *S. parasanguinis* and *S. equinus* genomes are predicted to encode β–glucosidases (EC 3.2.1.21) that are required for the conversion of cellobiose to β–D–glucose (figure S3). However, only *S. equinus* is able to ferment cellobiose [12], which appears to be in agreement with the presence of genes encoding a complete cellobiose PTS, which was exclusively encountered in the *S. equinus* genome (Figure 4).

These genome-based predictions are in good agreement with the differential carbohydrate-fermentation and growth patterns that were previously determined [12]. However, all *S. salivarius* genomes lack the PTS for lactose, while all strains were able to ferment this substrate [12]. It is known that *S. salivarius*, and its close relative *S. thermophilus*, can effectively ferment lactose and import this substrate by a dedicated lactose permease (LacS) that belongs to the galactoside-pentose-hexuronide translocator family [70], [71]. Indeed, all *S. salivarius*, and *S. equinus* genomes presented here appeared to encode a *lacS* homologue. The *S. parasanguinis* strain also encode a complete PTS EII complex predicted to be involved in import of galactose and galactosamine. To metabolize lactose and its galactose moiety, *S. parasanguinis* and *S. equinus* encode the tagatose phosphate and/or Leloir pathways [72]. The *S. salivarius* lineages, lacking the PTS for lactose, relied on the latter pathway for metabolism of these substrates (Figure S3).

All sequenced genomes encode maltose/maltodextrin ABC transporters, while *S. parasanguinis* and *S. equinus* also appear to encode ABC transporters for multiple sugars (the so-called MSM system). These transporters have been previously described for *S. mutans* and can import multiple sugars, including raffinose and melibiose [73]. Notably, the *S. parasanguinis* and *S. equinus* strains, also encode the downstream enzymes required for raffinose and melibiose metabolism. Finally, the *S. equinus* genome also contained genes encoding an “α-arabinosides ABC transport permease (araP)” as well as the necessary enzymes to metabolize this sugar. These findings are in excellent agreement with the observation that only *S. equinus* was able to grow on arabinose, while only *S. parasanguinis* and *S. equinus* could ferment melibiose and grow on raffinose [12].

Though all *Streptococcus* strains encoded at least one α-amylase, *S. equinus* could grow on media with starch as the sole carbohydrate source [12]. However, this could be explained by the predicted subcellular location of the α-amylase enzymes. The enzymes encoded by *S. parasanguinis* and *S. salivarius* genomes were all predicted to be cytoplasmic, while only the *S. equinus* strain appeared to encode an excreted α-amylase (as well as a cytoplasmic one). Thereby, it seems likely that only *S. equinus* can access extracellular starch as a substrate for growth, whereas *S. parasanguinis* and the *S. salivarius* strains may use intracellular polysaccharides (IPS) for energy-storage. The latter is supported by the fact the *S. parasanguinis* and *S. salivarius* strains encoded three key enzymes required for IPS synthesis, namely glycogen synthase (EC 2.4.1.21), glucose-1-phosphate adenylyltransferase (EC 2.7.7.27), and branching enzyme (EC 2.4.1.18) [74]. However, to the best of our knowledge there is no experimental evidence to support the capacity for IPS synthesis in any of the streptococcal species studied here, which may suggest that this only occurs under specific circumstances that were not studied to date.

Since the observed physiological characteristics of the *Streptococcus* strains are in excellent agreement with genome predictions [12], the small-intestinal *Streptococcus* genomes are helpful to predict the effect of dietary changes on the *Streptococcus* populations in the small-intestine.

### Detection of small-intestinal streptococcal strains and lineages

To monitor the dynamics of the small-intestinal *Streptococcus* populations during dietary intervention studies, fast and high throughput, PCR-based detection assays were developed that targeted genes that were solely encountered in a single small-intestinal *Streptococcus* strain or lineage.

To evaluate the specificity and conservation of the genes selected for detection among the representative isolates that belong to the different streptococcal lineages, they were amplified from 92 *Streptococcus* isolates and 28 *Enterococcus* isolates as negative control [12]. The PCR amplicons robustly discriminated the 92 *Streptococcus* isolates into 3 *S. parasanguinis* isolates, 13 *S. equinus* isolates, 1 *S. salivarius* lineage 2 isolate, and 8 *S. salivarius* lineage 3 isolates, thereby perfectly matching with their grouping according to AFLP and Rep-PCR (Table 2 and S12; [12]). These results showed that among representative strains of the identified lineages, the selected gene is conserved and specific for the isolates of that lineage. In contrast, the primers designed to selectively amplify *S. salivarius* strain 1 and 4, failed to consistently discriminate between isolates of these two lineages, albeit that not every bacterial isolate belonging to *S. salivarius* lineage 4 revealed a PCR product with primers for *S. salivarius* lineage 1. Nonetheless, these results confirm the close relatedness of these lineages, which was already apparent from the identical Rep-PCR profiles they generated (see above) [12]. Furthermore, the *Streptococcus* PCR assays developed here provide a simple and effective means to detect the small-intestinal *S. parasanguinis, S. equinus,* and *S. salivarius* lineages 2, 3 and the group of *S. salivarius* lineage 1 and 4.

**Table 2:** Number of positive PCR amplifications of bacterial isolates with *Streptococcus* lineage specific primers.

Grouping		*S. parasanguinis*	*S. equinus*	*S. salivarius*				*Enterococcus*
				1	2	3	4	
AFLP and Rep-PCR analysis*		3	13	1	1	8	66	28
PCR assay	*S. parasanguinis*	3	-	-	-	-	-	-
	*S. equinus*	-	13	-	-	-	-	-
	*S. salivarius* 1	-	-	1	-	-	56	-
	*S. salivarius* 2	-	-	-	1	-	-	-
	*S. salivarius* 3	-	-	-	-	8	-	-
	*S. salivarius* 4	-	-	1	-	-	66	-

: Results from grouping according to AFLP and Rep-PCR [12].

Considering that all six *Streptococcus* strains from the small-intestine were cultivated from a single ileostoma effluent sample, the PCR assays were further evaluated with total DNA from 30 ileostoma effluent samples obtained from 6 ileostomists, 4 ileal fluid samples from 3 healthy individuals, and fecal samples from 10 healthy individuals to investigate the distribution of these genetic targets beyond the ileostomist they were derived from. As anticipated, the selected genes from all sequenced *Streptococcus* genomes were amplified in other ileostoma effluent samples collected from the ileostomist from which the strains were isolated (Subject A; Table 3). However, the *S. equinus* targeted amplicon could only be detected in a single ileostoma effluent sample (Subject A) and in several fecal samples (Table 3). The *S. parasanguinis* targeted amplicon was exclusively detected in ileostoma samples, which were obtained from subject A and subject B. The latter samples were collected on two consecutive days, while additional samples that were collected from the same individual 5 years later did not allow the detection of this genetic marker. Considering that the *S. parasanguinis* and, to a lesser extent, *S. equinus* belong to the predominant streptococci in the small-intestine in these samples [12], these findings imply that the small-intestinal microbiota in other individuals is encompassing other *S. parasanguinis* and *S. equinus* lineages as compared to the strain targeted here. In contrast, the unique genes from at least 2 *S. salivarius* lineages (mostly lineage 2 and 4) were detected in all but one ileostoma effluent sample, all ileal fluid samples as well as several fecal samples obtained from other individuals (Table 3). These findings suggest that the *S. salivarius* lineages are highly conserved in the small-intestine and, to a lesser extent in the terminal part of the gastrointestinal tract, among the different subjects.

**Table 3:** Detection of *S. parasanguinis*, *S. equinus* and *S. salivarius* genetic targets in intestinal samples.

Subject		Year	Day	Time of sampling	*S. parasanguinis*	*S. equinus*	*S. salivarius*			
							1	2	3	4
Ileostoma effluent	A*	1	1	Morning	D	-	-	-	D	D
				Afternoon	D	-	-	D	D	D
			4	Morning	D	-	-	-	D	D
				Afternoon	D	-	-	-	D	D
			7	Morning	D	-	-	-	D	D
				Afternoon	D	-	-	-	D	D
			9	Morning	D	-	-	-	D	D
				Afternoon	D	-	-	-	D	D
		6	1	Afternoon	D	D	D	D	D	D
			3	Morning	-	-	-	-	-	-
				Afternoon	D	-	D	D	D	D
	B	1	1	Afternoon	D	-	-	-	-	D
			2	Afternoon	D	-	-	D	-	D
		6	1	Morning	-	-	D	-	D	D
			3	Morning	-	-	D	-	D	D
				Afternoon	-	-	D	D	D	D
	C	1	1	Afternoon	-	-	-	D	D	D
			2	Afternoon	-	-	-	D	D	D
		6	1	Morning	-	-	D	D	-	D
				Afternoon	-	-	D	D	-	D
			3	Morning	-	-	D	D	-	D
				Afternoon	-	-	-	D	-	D
	D	1	1	Afternoon	-	-	-	D	-	D
			2	Afternoon	-	-	-	D	-	D
	E	1	1	Afternoon	-	-	-	D	-	D
			2	Afternoon	-	-	-	D	-	D
	F	6	1	Morning	-	-	D	D	-	D
				Afternoon	-	-	D	D	-	D
			3	Morning	-	-	D	D	-	-
				Afternoon	-	-	D	D	-	D
Small intestinal fluid	G (ileum)				-	-	-	D	-	D
	H (jejunum)				-	-	D	D	-	D
	H (ileum)				-	-	D	D	-	D
	I (ileum)				-	-	-	D	D	-
Fecal sample	J				-	D	ND	D	-	D
	K				-	-	ND	-	-	-
	L				-	-	ND	-	-	-
	M				-	-	ND	D	-	-
	N				-	D	ND	D	-	D
	O				-	-	ND	-	-	-
	P				-	-	ND	D	-	D
	Q				-	-	ND	-	-	-
	R				-	-	ND	-	-	-
	S				-	D	ND	D	-	D

: Ileostomist from which sequenced *Streptococcus* strains were obtained.

D: Detected; ND: Not determined.

## Discussion

Streptococci are common colonizers of the human small-intestine and are important in metabolic conversion of diet-derived carbohydrates that are present in this ecosystem [11], [12]. To further our knowledge of the environmental interaction-potential and the metabolic capacity, the genomes of six small-intestinal *Streptococcus* isolates were determined by next generation sequencing technologies and were compared with *Streptococcus* genomes from the public databases.

Phylogenetic analysis of the small-intestinal *Streptococcus* genomes placed one strain into the *S. mitis* species-group, one strain into the *S. bovis* species-group, and 4 into the *S. salivarius* species-group, matching species identifications that were previously based on the 16S rRNA gene sequence alone [12]. The genomes of two of the *S. salivarius* strains (lineage 1 and 4) were highly similar, which was expected based on their highly similar genetic typing profiles and their conserved physiological characteristics [12].

The *Streptococcus* pangenome consisted of 12,403 orthologous DNA sequences, which is double the size predicted by Lefébure and Stanhope based on 26 *Streptococcus* finished and whole genome shotgun genomes [42]. However, this genome set was represented by 6 species while the current study included as many as 20 different species. The core *Streptococcus* genome was defined here as a set of 574 OG shared by all *Streptococcus* genomes, which is in line with earlier predictions [42]. Analysis of the core *Streptococcus* OG revealed that the function of most genes was well defined and belonged to typically conserved cellular processes like transcription, translation and replication. Nonetheless, a significant portion of core orthologous groups were involved in metabolism, especially transport and metabolism of nucleotides and carbohydrates. Likewise, a group of 197 OG that in the current analysis are only shared among the streptococci analysed here was predominated by OG involved in metabolism and may represent functions that contribute to the lifestyle of these bacteria in the (human small) intestine.

Mining of the genomes revealed that the small-intestinal streptococci coded for two-component regulatory modules, such as those involved in natural competence. Since natural competence is a mediator for evolution and genomic plasticity [27], we focused on functions that play an important role in this system. We found gene repertoires that imply that the competence regulon in the streptococcal genomes analysed here is complete. Moreover, the regulatory circuits involved in controlling expression of the competence genes contain conserved components in the *S. salivarius* genomes, including the communication peptide pheromone and their cognate receptors. These appeared to be identical for strain HSISS1 and 4, but were distinct from the matching peptides found for HSISS2 and 3). This finding suggests that within the small-intestinal habitat these strains could activate competence via inter-strain crosstalk, thereby stimulating genetic exchange between streptococcal members of this ecosystem. Furthermore, mobile elements were identified in the small-intestinal *Streptococcus* genomes, testifying that there is potential for the streptococci to engage in horizontal transfer of genes.

The genomes of the small-intestinal *Streptococcus* strains encoded the capacity to synthesize a large number of amino acids, as well as a number of B-vitamins. As expected, all streptococcal genomes analysed here encoded a complete glycolytic pathway and a (partially) intact pentose phosphate pathway for energy generation, yielding lactate, acetate, formate, and possibly acetoin as fermentation products. However, the strains differed considerably in their predicted capacity to transport and metabolize specific sugars. The *Streptococcus* genomes encoded a complement of 11 different complete PTS, which in some cases were present in multiple copies in a single strain. This could indicate that some substrates may be more important for certain strains. The *S. equinus* genome encoded for 9 different PTS, which was higher compared to the number of PTS encoded by *S. parasanguinis* (8) and the *S. salivarius* strains (4−6). In addition, this strain was also the only sequenced small-intestinal strain that appeared to encode transporters for arabinose and extracellular amylases for the degradation of starch. Nevertheless, the number of PTS was relatively low compared to a closely related strain *S. gallolyticus* UCN34, which encodes 25 PTS [75].

While the *S. equinus* strain encoded for extra- and intracellular α-amylases, the *S. parasanguinis* and *S. salivarius* strains were found to only code for those that remain intracellular, which have been postulated to play a role in breakdown of IPS [76]. However, investigations into α-amylase of *S. mutans* revealed that intracellular α-amylase was not essential for breakdown of IPS and dextrins from starch digested by exogenous α-amylase [77]. Therefore, the role of intracellular α-amylases remains to be elucidated.

The encoded carbohydrate transporters, and the reconstructions of the metabolic pathways based on genome analysis were in excellent agreement with physiological characteristics that were determined previously [12]. The variation between their metabolic capacities may explain their dynamic abundance in a harsh and fluctuating environment such as the small-intestine [12] (Leimena and Van den Bogert, et al., Unpublished data). As streptococci are fast-growing and efficient fermenters of simple carbohydrates [11] the combined metabolic capacity of the small-intestinal *Streptococcus* population may make a considerable contribution to the primary digestion of food components in this ecosystem that competes with that of the host [11]. The streptococcal fermentation products (e.g. lactate and acetate) may serve as an energy source for the intestinal mucosa. In addition, the short chain fatty acids support growth of secondary fermenters in the small intestinal ecosystem, including *Veillonella* that together with streptococci have a potential to form a food-chain relationship [11] as well as members of the genus *Clostridium* that produce butyrate from acetate (Leimena and Van den Bogert, et al., Unpublished data).

The concordance between genome-based metabolic pathways and physiological characteristtics suggests that the small-intestinal genomes are useful in the prediction of the carbohydrate utilization capacities of these bacterial strains. This predictive value of the *Streptococcus* genomes presented here may be of use in studies that aim to determine the effect of food components on the small-intestinal microbiota *in situ* with a special focus on these *Streptococcus* populations. One prerequisite to this concept is the capacity to effectively detect the *Streptococcus* lineages, using for example unique genes as genetic markers, in intestinal samples. To this end, PCR-based screening assays were designed for each of the small-intestinal streptococci and tested with 92 *Streptococcus* isolates. These assays correctly amplified isolates belonging to the same lineage as their target *Streptococcus* strain, based on strain-level groupings as was done with AFLP and Rep-PCR analysis [12]. Although primer assays for *S. salivarius* lineage 1 and 4 isolates were developed using genes that were not encountered in other genomes and strict primer design parameters to ensure primer specificity, both primer sets showed cross-reaction with isolates belonging to the non-target *S. salivarius* lineage. Determining the exact causes for this is not trivial and are likely related to the reasons underlying the inaccurate estimation of strain-specific genes (Table S7). Nonetheless, the *Streptococcus* PCR assays developed here provide a simple and rapid method for the screening of large numbers of samples from, for example, dietary intervention studies, for the genes that were exclusively encountered in the genomes of the small-intestinal *S. parasanguinis, S. equinus,* and *S. salivarius* strains or lineages.

Application of the assays on 34 intestinal and 10 fecal samples collected from 19 human individuals revealed that at least two *S. salivarius* lineages were present in almost all small-intestinal samples and several fecal samples, indicating that these strains are common colonizers and represent an important population of, in particular, the small-intestinal microbiota. Only one ileostoma effluent sample showed no amplification within any of the assays. However, the *Streptococcus* population in this sample is most likely represented by one or more *Streptococcus* strains that do not carry the unique genes targeted by PCR-based detection assays.

In conclusion, the work presented here describes a comparative genomics study of *Streptococcus* spp. that focused on strains from the human small-intestine. Comparative genomic analysis revealed that the small-intestinal strains differed in their predicted transport and metabolism of sugars, which was in agreement with physiological data. Therefore, the small-intestinal *Streptococcus* genomes are useful to construct metabolic models to predict the effect of different dietary substances on *Streptococcus* population dynamics in the human small-intestine. Furthermore, assays designed for detection of two *S. salivarius* strains were positive for most of the small-intestinal samples from different individuals, suggesting that strains, carrying the target functional gene, represent an important population of the small-intestinal ecosystem.

## Supporting Information

Figure S1 **Multiple alignment of protein sequences from transposases identified in small-intestinal** ***Streptococcus*** **genomes.**
(EPS)

Figure S2 **Pathways for amino acid metabolism identified in small-intestinal** ***Streptococcus*** **genomes.**
(EPS)

Figure S3 **Metabolic pathways for sugar metabolism identified in small-intestinal** ***Streptococcus*** **genomes.**
(EPS)

Table S1 **Genome statistics for small-intestinal** ***Streptococcus*** **after scaffolding.**
(DOCX)

Table S2 **Characteristics of finished** ***Streptococcus*** genomes*.
(DOCX)

Table S3 **Primers used is this study.**
(DOCX)

Table S4 **Locus tags from 197 orthologous groups represented in 2-6 small-intestinal genomes.**
(XLSX)

Table S5 **Cluster specific orthologous groups.**
(XLSX)

Table S6 **Number of shared and unshared orthologous genes between** ***S. salivarius*** **genomic lineages 1-4.**
(DOCX)

Table S7 **Potential causes for inaccurate estimation of specific orthologous genes.**
(DOCX)

Table S8 **Number and description of two component systems predicted for small-intestinal** ***Streptococcus*** **strains.**
(DOCX)

Table S9 BOX, RUP, and SPRITE repeats found small-intestinal genomes*.
(DOCX)

Table S10 **Candidate bacteriocins identified by BAGEL2.**
(DOCX)

Table S11 **Locus tags of enzymes involved in glycolysis and pentose phosphate pathway.**
(DOCX)

Table S12 **Comparison of isolate groupings from genetic fingerprinting and results from lineage-specific PCRs.**
(DOCX)
