# Supplementary material for: Comparative Genomics Analysis of Streptococcus Isolates from the Human Small Intestine Reveals their Adaptation to a Highly Dynamic Ecosystem
Source: PLoS One. 2013 Dec 30;8(12):e83418. doi: 10.1371/journal.pone.0083418 (PMC3875467; doi:10.1371/journal.pone.0083418)
Supplement: Table S1 — Genome statistics for small-intestinal Streptococcus after scaffolding. (DOCX) [file pone.0083418.s004.docx]

Table S1: Genome statistics for small intestinal *Streptococcus*

|  | *S. parasanguinis* | *S. equinus* | *S. salivarius* | | | |
| --- | --- | --- | --- | --- | --- | --- |
|  |  |  | 1 | 2 | 3 | 4 |
| Locus tag prefix | HSISM1 | HSISB1 | HSISS1 | HSISS2 | HSISS3 | HSISS4 |
| Accession* | ASKI00000000 | ASKA00000000 | ASKB00000000 | ASKC00000000 | ASKH00000000 | ASKD00000000 |
| Total number of Contigs | 118 | 42 | 41 | 170 | 61 | 151 |
| Contig Sum (bp) | 2,219,316 | 1,864,835 | 2,074,878 | 2,103,121 | 2,218,190 | 2,034,088 |
| Max contig size (bp) | 7,5679 | 228,131 | 251,655 | 88,297 | 185,834 | 79,375 |
| Min contig size (bp) | 1,129 | 1,086 | 1,135 | 1,046 | 1,187 | 1,004 |
| Average contig size (bp) | 18,807 | 44,400 | 50,606 | 12,371 | 36,363 | 13,470 |
| contig N50 | 26,412 | 101,212 | 86,157 | 23,692 | 62,198 | 23,226 |
| Total number of scaffolds | 7 | 6 | 6 | 15 | 8 | 7 |
| Scaffold Sum (bp) | 2,334,368 | 1,906,608 | 2,127,962 | 2,360,589 | 2,399,642 | 2,208,371 |
| Max scaffold size (bp) | 1,227,939 | 1,486,018 | 1,616,043 | 702,120 | 1,081,630 | 957,480 |
| Min scaffold size (bp) | 3,313 | 9,467 | 1,135 | 1,217 | 43,206 | 1,004 |
| Average scaffold size (bp) | 333,481 | 317,768 | 354,660 | 157,372 | 299,955 | 315,481 |
| Scaffold N50 | 1,227,939 | 1,486,018 | 1,616,043 | 259,673 | 516,471 | 763,578 |
| GC content (%) | 41.45 | 37.24 | 40.12 | 39.84 | 39.31 | 40.23 |
| Number of predicted proteins | 2,226 | 2,160 | 1,939 | 2,247 | 2,230 | 2,091 |
| Genes assigned to COG | 1828 | 1928 | 1709 | 1862 | 1800 | 1828 |
| Genes assigned to KEGG | 1094 | 1117 | 1106 | 1082 | 1110 | 1086 |

*: The version described in this paper is version XXXX0100000
