# Supplementary material for: Comparative Genomics Analysis of Streptococcus Isolates from the Human Small Intestine Reveals their Adaptation to a Highly Dynamic Ecosystem
Source: PLoS One. 2013 Dec 30;8(12):e83418. doi: 10.1371/journal.pone.0083418 (PMC3875467; doi:10.1371/journal.pone.0083418)
Supplement: Table S2 — Characteristics of finished Streptococcus genomes*. (DOCX) [file pone.0083418.s005.docx]

Table S2: Characteristics of finished *Streptococcus* genomes*

| Accession | Organism | Goldcard | Isolation site | Host | Comments |
| --- | --- | --- | --- | --- | --- |
| NC_002737 | *Streptococcus pyogenes* M1 GAS | Gc00049 | Patient with a wound infection | Homo sapiens |  |
| NC_003028 | *Streptococcus pneumoniae* TIGR4 | Gc00058 | Blood of a 30 year old male patient in Kongsvinger Norway | Homo sapiens |  |
| NC_003098 | *Streptococcus pneumoniae* R6 | Gc00065 | - | Homo sapiens |  |
| NC_003485 | *Streptococcus pyogenes* MGAS8232 | Gc00081 | throat swab from patient with acute rheumatic fever | Homo sapiens |  |
| NC_004070 | *Streptococcus pyogenes* MGAS315 | Gc00094 | patient with streptococcal toxic shock syndrome | Homo sapiens |  |
| NC_004116 | *Streptococcus agalactiae* 2603V/R | GC00098 | clinical isolate | Homo sapiens |  |
| NC_004350 | *Streptococcus mutans* UA159 | GC00109 | Child with active dental caries in 1982 | Homo sapiens |  |
| NC_004368 | *Streptococcus agalactiae* NEM316 | Gc00100 | Case of fatal septicemia | Homo sapiens |  |
| NC_004606 | *Streptococcus pyogenes* SSI-1 | Gc00137 | Toxic-shock patient in Japan | Homo sapiens |  |
| NC_006086 | *Streptococcus pyogenes* MGAS10394 | Gc00205 | pharyngeal swab from child, during a study of the epidemiology of pharyngitis in a private elementary school | Homo sapiens |  |
| NC_006448 | *Streptococcus thermophilus* LMG 18311 | Gc00234 | Commercial yogurt in 1974 in the United Kingdom | - |  |
| NC_006449 | *Streptococcus thermophilus* CNRZ1066 | Gc00233 | Isolated from yogurt in France | - |  |
| NC_007296 | *Streptococcus pyogenes* MGAS6180 | Gc00284 | Invasive disease in Texas in 1998 | Homo sapiens |  |
| NC_007297 | *Streptococcus pyogenes* MGAS5005 | Gc00285 | Invasive case in Ontario | Homo sapiens |  |
| NC_007432 | *Streptococcus agalactiae* A909 | Gc00302 | septic human neonate | Homo sapiens |  |
| NC_008021 | *Streptococcus pyogenes* MGAS9429 | Gc00379 | pharyngeal swab from animal | Homo sapiens | swab from animal but host name was still homo sapiens |
| NC_008022 | *Streptococcus pyogenes* MGAS10270 | GC00378 | pharyngeal swab from child | Homo sapiens |  |
| NC_008023 | *Streptococcus pyogenes* MGAS2096 | Gc00377 | patient with acute poststreptococcal glomerulonephritis | Homo sapiens |  |
| NC_008024 | *Streptococcus pyogenes* MGAS10750 | Gc00376 | human pharyngeal swab from patient with pharyngitis | Homo sapiens |  |
| NC_008532 | *Streptococcus thermophilus* LMD-9 | Gc00451 | - | - |  |
| NC_008533 | *Streptococcus pneumoniae* D39 | Gc00437 | - | Homo sapiens |  |
| NC_009009 | *Streptococcus sanguinis* SK36 | Gc00509 | isolated from human dental plaque | Homo sapiens |  |
| NC_009332 | *Streptococcus pyogenes* str. Manfredo | Gc00455 | Patient in the 1950's in Chicago | Homo sapiens |  |
| NC_009442 | *Streptococcus suis* 05ZYH33 | Gc00546 | Chinese virulent strain isolated from fatal cases of STSS in 2005 | Homo sapiens |  |
| NC_009443 | *Streptococcus suis* 98HAH33 | Gc00547 | Chinese virulent strain isolated from fatal cases of STSS in 1998 | Homo sapiens |  |
| NC_009785 | *Streptococcus gordonii* str. Challis substr. CH1 | Gc00643 | - | Homo sapiens |  |
| NC_010380 | *Streptococcus pneumoniae* Hungary19A-6 | Gc00735 | Human ear, Hungary | Homo sapiens |  |
| NC_010582 | *Streptococcus pneumoniae* CGSP14 | Gc00765 | clinical isolate derived from a child with necrotizing pneumonia, simultaneously complicated with HUS, at Chang Gung Memorial Hospital and Children's Hospital, Taoyuan, Taiwan. | Homo sapiens |  |
| NC_011072 | *Streptococcus pneumoniae* G54 | Gc00837 | Genova Italy by G. Schito from a respiratory sample in 1997 | Homo sapiens |  |
| NC_011134 | *Streptococcus equi* subsp. *zooepidemicus* MGCS10565 | Gc00845 | throat of a patient with nephritis diagnosed during an epidemic in the state of Minas Gerais, Brazil | Homo sapiens |  |
| NC_011375 | *Streptococcus pyogenes* NZ131 | Gc00871 | Patient with acute glomerulonephritis and was provided by Diana Martin, New Zealand Communicable Diseases Center, Porirua, New Zealand | Homo sapiens |  |
| NC_011900 | *Streptococcus pneumoniae* ATCC 700669 | Gc00940 | hospital, Barcelona, Spain | Homo sapiens |  |
| NC_012004 | *Streptococcus uberis* 0140J | Gc00948 | clinical bovine mastitis case | Bovine |  |
| NC_012466 | *Streptococcus pneumoniae* JJA | Gc00973 | - | Homo sapiens |  |
| NC_012467 | *Streptococcus pneumoniae* P1031 | Gc00972 | - | Homo sapiens |  |
| NC_012468 | *Streptococcus pneumoniae* 70585 | Gc00969 | - | Homo sapiens |  |
| NC_012469 | *Streptococcus pneumoniae* Taiwan19F-14 | Gc00974 | cerebrospinal fluid, Taiwan | Homo sapiens |  |
| NC_012470 | *Streptococcus equi* subsp. *zooepidemicus* |  |  |  | No GOLDCARD available, genbank entry: nasal swab taken from a healthy Thoroughbred racehorse |
| NC_012471 | *Streptococcus equi* subsp. *equi* 4047 | Gc00971 | horse with strangles from New Forest, UK | Horse |  |
| NC_012891 | *Streptococcus dysgalactiae* subsp. *equisimilis* GGS_124 | Gc01051 | patients with STSS | Homo sapiens |  |
| NC_012924 | *Streptococcus suis* SC84 | Gc01063 | case of streptococcal toxic shock-like syndrome in Sichuan Province, China in 2005 | Homo sapiens |  |
| NC_012925 | *Streptococcus suis* P1/7 | Gc01061 | ante-mortem blood culture from a pig dying with meningitis | Sus scrofa, Homo sapiens |  |
| NC_012926 | *Streptococcus suis* BM407 | Gc01062 | CSF from a human case of meningitis in Ho Chi Minh City, Vietnam in 2004 | Homo sapiens |  |
| NC_013798 | *Streptococcus gallolyticus* UCN34 |  |  |  | No GOLDCARD available |
| NC_013853 | *Streptococcus mitis* B6 | Gc01214 | hospital in Bochum, Germany | Homo sapiens |  |
| NC_013928 | *Streptococcus mutans* NN2025 | Gc01074 | clinical serotype c strain isolated in Japan in 2002 from a patient with dental caries | Homo sapiens |  |
| NC_014251 | *Streptococcus pneumoniae* TCH8431/19A | Gc01351 | respiratory tract | Homo sapiens |  |
| NC_014494 | *Streptococcus pneumoniae* AP200 | Gc01425 | clinical isolate from the cerebrospinal fluid of a patient with meningitis in Italy in 2003 | Homo sapiens |  |
| NC_014498 | *Streptococcus pneumoniae* 670-6B | Gc01424 | - | Homo sapiens |  |
| NC_015215 | *Streptococcus gallolyticus* subsp. *gallolyticus* ATCC BAA-2069 | Gc01837 | human blood culture; infective endocarditis | Homo sapiens |  |
| NC_015291 | *Streptococcus* *oralis* Uo5 | Gc01712 | human mouth | Homo sapiens |  |
| NC_015433 | *Streptococcus* *suis* ST3 | Gc01735 | - | Homo sapiens, Sus scrofa |  |
| NC_015558 | *Streptococcus* *parauberis* KCTC 11537 | Gc01743 | - | - |  |
| NC_015600 | *Streptococcus* *pasteurianus* ATCC 43144 | Gc01797 | human blood | Homo sapiens |  |
| NC_015678 | *Streptococcus* *parasanguinis* ATCC 15912 | Gc01842 | human throat | Homo sapiens |  |
| NC_015760 | *Streptococcus* *salivarius* CCHSS3 | Gc01887 | Human blood | Homo sapiens |  |
| NC_015875 | *Streptococcus* *pseudopneumoniae* IS7493 | Gc01960 | sputum of a patient with human immunodeficiency virus (HIV) who had documented pneumonia | Homo sapiens |  |
| NC_016749 | *Streptococcus* *macedonicus* ACA-DC 198 | Gc02096 | traditional greek kasseri cheese | - |  |

*: Retrieved from the Genome OnLine database (GOLD; <http://genomesonline.org>) on February 27^th^, 2012
