# Supplementary material for: Comparative Genomics Analysis of Streptococcus Isolates from the Human Small Intestine Reveals their Adaptation to a Highly Dynamic Ecosystem
Source: PLoS One. 2013 Dec 30;8(12):e83418. doi: 10.1371/journal.pone.0083418 (PMC3875467; doi:10.1371/journal.pone.0083418)
Supplement: Table S3 — Primers used is this study. (DOCX) [file pone.0083418.s006.docx]

Table S3: Primers used is this study

| **Primer name*** | **Primer sequence (5'-3')** | **Annotation of target gene** | **Expected PCR product** |
| --- | --- | --- | --- |
| HSISM1_486_fwd | CGCTGCTTTAGAGGCATCAACCG | Hypothetical protein | 225 |
| HSISM1_486_rev | ACCTGGCGATCAAGCACAGAGT |  |  |
| HSISB1_163_fwd | ACTTTGGTGCGTTATCCTGGTGG | Alpha-N-arabinofuranosidase (EC 3.2.1.55) | 230 |
| HSISB1_163_rev | TCAACTAAATGGCGGGCTTCGTC |  |  |
| HSISS1 _1164_fwd | ACTGGTTGTTCTGGCTCCTCTGG | Hypothetical protein | 223 |
| HSISS1_1164_rev | CGGTCGTACCAGATGTACCAGGC |  |  |
| HSISS2_1351_fwd | GGTTGGCTTGGTTCTTTACGGGT | Hypothetical protein | 230 |
| HSISS2_1351_rev | GGCTCCAAAGCTCGAATGGTTGC |  |  |
| HSISS3_521_fwd | GCTGAACCAACAAACCTCGCAGA | Hypothetical protein; possible cell wallprotein, WapE | 286 |
| HSISS3_521_rev | TGGCAACCTCTTGGTCGAGTGCT |  |  |
| HSISS4_733_fwd | GGCTGAACCTGATCCTCCATTCG | Hypothetical protein | 170 |
| HSISS4_733_rev | AACGAGACGAGTCAAAGGGCTTG |  |  |

*: Target strain_locus tag_fwd/rev

Fwd: forward primer, rev: reverse primer, HSISM1 represents the small-intestinal *S. parasanguinis* genome (*S. mitis* species group), HSISB1 represents the small-intestinal *S. equinus* genome (*S. bovis* species group), and HSISS1, HSISS2, HSISS3, and HSISS4 represents the small-intestinal *S. salivarius* genomes from lineage 1, 2, 3, and 4 (*S. salivarius* species group), respectively.
