# Supplementary material for: Comparative Genomics Analysis of Streptococcus Isolates from the Human Small Intestine Reveals their Adaptation to a Highly Dynamic Ecosystem
Source: PLoS One. 2013 Dec 30;8(12):e83418. doi: 10.1371/journal.pone.0083418 (PMC3875467; doi:10.1371/journal.pone.0083418)
Supplement: Table S6 — Number of shared and unshared orthologous genes between S. salivarius genomic lineages 1-4. (DOCX) [file pone.0083418.s009.docx]

Table S6: Number of shared (lower left panel) and unshared (upper right panel) orthologous genes between *S. salivarius* genomic lineages 1-4

|  | Lineage 1 | Lineage 2 | Lineage 3 | Lineage 4 |
| --- | --- | --- | --- | --- |
| Lineage 1 | 1858^b^ | 570 ^c^  311^c^ | 433 ^c^  263 ^c^ | 237 ^c^  128 ^c^ |
| Lineage 2 | 1547^a^  (76) | 2117^b^ | 398 ^c^  487 ^c^ | 397 ^c^  547 ^c^ |
| Lineage 3 | 1595^a^  (124) | 1630^a^  (159) | 2028^b^ | 415 ^c^  476 ^c^ |
| Lineage 4 | 1730^a^  (259) | 1570^a^  (99) | 1552^a^  (81) | 1967^b^ |

^a^: numbers in brackets indicate number of shared orthologous genes between two strains without number of shared genes among all *S. salivarius* strains (1471)

^b^: total number of orthologous groups per lineage

^c^: number of strain-specific OG for the *S. salivarius* strain specified in the row (lower left of the cell) and in the column (upper right of the cell) when both strains are compared.
