# Supplementary material for: Comparative Genomics Analysis of Streptococcus Isolates from the Human Small Intestine Reveals their Adaptation to a Highly Dynamic Ecosystem
Source: PLoS One. 2013 Dec 30;8(12):e83418. doi: 10.1371/journal.pone.0083418 (PMC3875467; doi:10.1371/journal.pone.0083418)
Supplement: Table S7 — Potential causes for inaccurate estimation of specific orthologous genes. (DOCX) [file pone.0083418.s010.docx]

Table S7: Potential causes for inaccurate estimation of strain specific orthologous genes between *S. salivarius* lineage 1 and 4^a^

| Number of genes from lineage 1 | Description |
| --- | --- |
| 61 | genes did not have an ortholog, but did contain one or more conserved regions potentially encoded by the lineage 4 genome (either as part of non-orthologous genes, or in a genomic region not predicted to encode protein, including gene fragments introduced by single nucleotide insertion or deletion in linage 4) |
| 42 | genes were N or C-terminal fragments of frameshifts in orthologous 4 lineage genes introduced by single-nucleotide insertions or deletions in lineage 1 |
| 12 | genes were very small (<60 amino acid residues) and had not been recognized as ORFs in lineage 4 |
| 8 | genes did not have a significantly similar sequence |
| 5 | genes were encoded in genomic regions for which only partial or no sequence data was available in lineage 4^b^ |

^a^: causes that may have led to an overestimation of the strain-specific OG prediction were determined by manual analyses of strain-specific ortholgous genes of the strain from *S. salivarius* lineage 1, employing OG-protein sequence detection by tblastn [[1](#_ENREF_1)] in the genome that was predicted to lack the orthologous genes (lineage 4).

^b^: prediction based on analysis of conserved gene context of neighboring genes

REFERENCES

1. Altschul SF, Gish W, Miller W, Myers EW, Lipman DJ (1990) Basic local alignment search tool. J Mol Biol 215: 403-410.
