# Supplementary material for: Comparative Genomics Analysis of Streptococcus Isolates from the Human Small Intestine Reveals their Adaptation to a Highly Dynamic Ecosystem
Source: PLoS One. 2013 Dec 30;8(12):e83418. doi: 10.1371/journal.pone.0083418 (PMC3875467; doi:10.1371/journal.pone.0083418)
Supplement: Table S8 — Number and description of two component systems predicted for small-intestinal Streptococcus strains. (DOCX) [file pone.0083418.s011.docx]

Table S8: Number and description of two component systems predicted for small intestinal *Streptococcus* strains.

|  | *S. parasanguinis* | *S. equinus* | *S. salivarius* | | | | Comment |
| --- | --- | --- | --- | --- | --- | --- | --- |
|  |  |  | 1 | 2 | 3 | 4 |  |
| Total number of two component systems | 14 | 13 | 14 | 12 | 18 | 14 |  |
| Number of orphan two component system^a^ | 4 | 0 | 3 | 3 | 1 | 3 |  |
| CiaRH | x | x | x | x | x | x | See main text |
| ComDE | x | x | x | x | x | x | See main text |
| VraSR | x | x | x | x | x | x | See main text |
| CsrSR | x^b^ | x | x | x | x | x | See main text |
| SA14-24 | x | x | x | x | x | x | To the best of our knowledge, no function for this TCS has been proposed |
| DltR |  | x |  |  |  |  | Involved in regulating incorporation of D-alanine in lipoteichoic acid [[1](#_ENREF_1)]. |
| RelRS |  |  | x | x | x | x | Involved in regulation of RelP (producing a (p)ppGpp synthase) [[2](#_ENREF_2)] |
| BlpH | x |  |  |  |  |  | Proposed sensor histidine kinase involved in regulation of genes with potential bacteriocin-like functions [[3](#_ENREF_3)]. |
| FasB |  |  |  |  |  | x | Sensor histidine kinase involved in control of Group A streptococcal virulence factors [[4](#_ENREF_4)]. |
| KdpD |  |  |  | x |  |  | Sensor histidine kinase that promotes resistance to osmotic, oxidative, and antimicrobial stress [[5](#_ENREF_5)]. |
| LevQRST | x | x |  |  |  |  | Four component system that consists of the histidine kinase LevS, the response regulator LevR, and two putative extracellular sugarbinding proteins (LevQ and LevT), controlling transcriptional regulation of *fruA* [[6](#_ENREF_6)]. |
| NisKR | x | x |  | x |  |  | The chromosomes of *S. parasanguinis*, *S. equinus*, and *S. salivarius* lineage 2 were predicted to code HK and RR controlling regulation of nisin biosynthesis by *L. lactis* (NisKR; [[7](#_ENREF_7),[8](#_ENREF_8)]). In addition, the *S. salivarius* strains also coded for TCS that was similar to SpaKR, which regulates subtilin biosynthesis by *Bacillus subtilis* [[9](#_ENREF_9)]. Both nisin and subtilin are lantibiotics and their gene clusters not only code for the nisin and subtilin precursors and proteins involved in their post-translational modification, but also contain genes involved in secretion of the modified precursors and immunity [[10](#_ENREF_10)]. However, the chromosomes of the newly sequenced *Streptococcus* strains did not appear to code for the complete nisin and subtilin biosynthetic gene clusters. These genes may have been deleted during evolution, indicating that the newly sequenced streptococci do not synthesize nisin and/or subtilin, or that the TCSs are involved in regulation of different systems. |
| SpaKR |  |  | x | x | x | x |  |
| VncRS | x |  | x |  |  | x | Two component system playing a role in induction of multiple pathways leading to cell death in *S. pneumonia* [[11](#_ENREF_11)]. However, this finding was challenged by a study by Haas, et al. showing that this TCS was not essential for cell death, so its function remains to be further elucidated [[12](#_ENREF_12)]. |
| YesMN | x |  |  |  |  |  | To the best of our knowledge, no function for this TCS has been proposed |

^a^: TCS component coding for either a sensor histidine kinase or response regulator

^b^: orphan response regulator

REFERENCES

1. Poyart C, Lamy MC, Boumaila C, Fiedler F, Trieu-Cuot P (2001) Regulation of D-alanyl-lipoteichoic acid biosynthesis in *Streptococcus agalactiae* involves a novel two-component regulatory system. J Bacteriol 183: 6324-6334.

2. Seaton K, Ahn SJ, Sagstetter AM, Burne RA (2011) A transcriptional regulator and ABC transporters link stress tolerance, (p)ppGpp, and genetic competence in Streptococcus mutans. J Bacteriol 193: 862-874.

3. de Saizieu A, Gardes C, Flint N, Wagner C, Kamber M, et al. (2000) Microarray-based identification of a novel *Streptococcus pneumoniae* regulon controlled by an autoinduced peptide. J Bacteriol 182: 4696-4703.

4. Kreikemeyer B, Boyle MD, Buttaro BA, Heinemann M, Podbielski A (2001) Group A streptococcal growth phase-associated virulence factor regulation by a novel operon (Fas) with homologies to two-component-type regulators requires a small RNA molecule. Mol Microbiol 39: 392-406.

5. Alegado RA, Chin CY, Monack DM, Tan MW (2011) The two-component sensor kinase KdpD is required for *Salmonella typhimurium* colonization of *Caenorhabditis elegans* and survival in macrophages. Cell Microbiol 13: 1618-1637.

6. Zeng L, Das S, Burne RA (2011) Genetic analysis of the functions and interactions of components of the LevQRST signal transduction complex of *Streptococcus mutans*. PLoS One 6: e17335.

7. Engelke G, Gutowski-Eckel Z, Kiesau P, Siegers K, Hammelmann M, et al. (1994) Regulation of nisin biosynthesis and immunity in *Lactococcus lactis* 6F3. Appl Environ Microbiol 60: 814-825.

8. Lubelski J, Rink R, Khusainov R, Moll GN, Kuipers OP (2008) Biosynthesis, immunity, regulation, mode of action and engineering of the model lantibiotic nisin. Cell Mol Life Sci 65: 455-476.

9. Klein C, Kaletta C, Entian KD (1993) Biosynthesis of the lantibiotic subtilin is regulated by a histidine kinase/response regulator system. Appl Environ Microbiol 59: 296-303.

10. Kleerebezem M (2004) Quorum sensing control of lantibiotic production; nisin and subtilin autoregulate their own biosynthesis. Peptides 25: 1405-1414.

11. Novak R, Charpentier E, Braun JS, Tuomanen E (2000) Signal transduction by a death signal peptide: uncovering the mechanism of bacterial killing by penicillin. Molecular cell 5: 49-57.

12. Haas W, Sublett J, Kaushal D, Tuomanen EI (2004) Revising the role of the pneumococcal vex-vncRS locus in vancomycin tolerance. Journal of bacteriology 186: 8463-8471.
