# Supplementary material for: Comparative Genomics Analysis of Streptococcus Isolates from the Human Small Intestine Reveals their Adaptation to a Highly Dynamic Ecosystem
Source: PLoS One. 2013 Dec 30;8(12):e83418. doi: 10.1371/journal.pone.0083418 (PMC3875467; doi:10.1371/journal.pone.0083418)
Supplement: Table S9 — BOX, RUP, and SPRITE repeats found small-intestinal genomes*. (DOCX) [file pone.0083418.s012.docx]

Table S9: BOX, RUP, and SPRITE repeats found small-intestinal genomes*

| Strain | | | *S. pneumoniae* | | | | | | | | *S. suis* | | | | | | | | | | | |
| --- | --- | --- | --- | --- | --- | --- | --- | --- | --- | --- | --- | --- | --- | --- | --- | --- | --- | --- | --- | --- | --- | --- |
|  |  |  | boxA | | boxB | | boxC | | SPRITE | | boxA | | boxB | | boxC | | boxD | | boxE | | boxF | |
|  |  |  | Score | E-value | Score | E-value | Score | E-value | Score | E-value | Score | E-value | Score | E-value | Score | E-value | Score | E-value | Score | E-value | Score | E-value |
| MSVId | HSISM1 | *Streptococcus parasanguinis* | 66.6 | 1.5e-19 | 38.7  32.1  25.9 | 3.5e-11  3.5e-09  2.5e-07 | 75.8 | 2.4e-22 | 107.8  103.6  57.4  44.1  28.2  27.1  9.7  7.8 | 5.8e-32  1e-30  8.2e-17  8.4e-13  5.4e-08  1.1e-07  0.02  0.072 | 25.2 | 4.3e-07 | 29.0  25.9  25.9  25.0 | 3e-08  2.6e-07  2.6e-07  4.8e-07 | 23.5  6.4  0.0 | 1.4e-06  0.18  8 | 0  5  1 | 7.9  0.49  5.4 | 20  17.3  10.1  5.4  29.1  14.4 | 1.60E-05  0.0001  0.015  0.38  2.70E-08  0.00076 |  |  |
| MSVIIe | HSISB1 | *Streptococcus equinus* |  |  |  |  |  |  |  |  | 3.1 | 1.6 | 12.7  4.8  -0.2 | 0.0025  0.57  8.5 |  |  | 1  0  20.6  1.2 | 5.3  8  9.7E-06  4.9 |  |  | 9.9 | 0.017 |
| MSIe | HSISS1 | *Streptococcus salivarius* | 48.7  11.5 | 3.6e-14  0.0055 | 66.7 | 1.3e-19 | 64.2  12.6 | 7.6e-19  0.0025 |  |  | 8.5 | 0.043 | -0.6 | 9.7 | 22.4  5.9 | 2.9e-06  0.26 | 8.5  0.1  1.4 | 0.046  7.8  4.5 |  |  |  |  |
| MSXVc | HSISS2 | *Streptococcus salivarius* |  |  | 40.4  27.2 | 1.1e-11  1e-07 |  |  |  |  |  |  | 3.6  -0.6 | 1.2  9.7 | 2.2 | 2.9 | 9.4  0.6  -0.3 | 0.024  6.3  8.9 | 6.8 | 0.14 |  |  |
| MSVIIf | HSISS3 | *Streptococcus salivarius* |  |  | 40.4 | 1.1e-11 |  |  |  |  |  |  | 8.4  -0.6 | 0.047  9.7 |  |  | 12  3.7  2.4  0.5  4.3  1.4  1.2 | 0.0038  1.2  2.5  6.7  0.75  4.3  4.9 |  |  |  |  |
| MSIIf | HSISS4 | *Streptococcus salivarius* | 48.7  11.5 | 3.6e-14  0.0055 | 66.7 | 1.3e-19 | 64.2  12.6 | 7.6e-19  0.0025 |  |  | 8.5 | 0.043 | -0.6 | 9.7 | 22.4  5.9 | 2.9e-06  0.26 | 0.3  0.1  8.5  1.4 | 7.2  7.8  0.046  4.5 |  |  |  |  |

* Repeats with values higher than the cut off score thresholds indicated by Croucher, et al. [[1](#_ENREF_1)] (for *S. pneumoniae* repeats: boxA: 30; boxB: 14; boxC: 28; RUP: 47; SPRITE: 66; for *S. suis* repeats: boxA: 60; BoxB 30; boxC: 30; boxD: 45; boxE: 80; boxF: 75) are highlighted in grey

The application of the Hidden Markov Models did not identify RUP repeats for any of the small-intestinal *Streptococcus* genomes

REFERENCES

1. Croucher NJ, Vernikos GS, Parkhill J, Bentley SD (2011) Identification, variation and transcription of pneumococcal repeat sequences. BMC Genomics 12: 120.
