# Supplementary material for: Comparative Genomics Analysis of Streptococcus Isolates from the Human Small Intestine Reveals their Adaptation to a Highly Dynamic Ecosystem
Source: PLoS One. 2013 Dec 30;8(12):e83418. doi: 10.1371/journal.pone.0083418 (PMC3875467; doi:10.1371/journal.pone.0083418)
Supplement: Table S10 — Candidate bacteriocins identified by BAGEL2. (DOCX) [file pone.0083418.s013.docx]

Table S10: Candidate bacteriocins identified by BAGEL2

| **Strain belonging to *Streptococcus* species group** | | **Locus tag** | **Peptide size**  **(amino acids)** | **Product** | **Class** | **Score*** |
| --- | --- | --- | --- | --- | --- | --- |
| *S. parasanguinis* | | HSISM1_1402 | 111 | Alkylphosphonate utilization operon proteinPhn A |  | 1150 |
|  |  | HSISM1_2084 | 105 | hypothetical protein |  | 1125 |
|  |  | HSISM1_1626 | 51 | hypothetical protein | IA | 1100 |
| *S. equinus* | | HSISB1_1269 | 101 | Endonuclease III (EC 4.2.99.18) |  | 1150 |
|  |  | HSISB1_1297 | 116 | Phenylalanyl-tRNA synthetase beta chain  (EC6.1 .1.20) |  | 1125 |
|  |  | HSISB1_1598 | 53 | hypothetical protein | IID | 6900 |
|  |  | HSISB1_1599 | 50 | hypothetical protein |  | 1125 |
| *S. salivarius* | 1 | HSISS1_1504 | 76 | hypothetical protein | IIA or IID | 6500 |
|  |  | HSISS1_1495 | 76 | Bacteriocin BlpU | IIA or IID | 6175 |
|  |  | HSISS1_1572 | 66 | hypothetical protein | IID | 1175 |
|  | 2 | HSISS2_2019 | 84 | pore-forming peptide, putative bacteriocin | IIA | 10100 |
|  |  | HSISS2_2023 | 76 | Bacteriocin BlpU | IIA | 5100 |
|  | 3 | HSISS3_415 | 84 | pore-forming peptide, putative bacteriocin | IIA or IID | 11150 |
|  |  | HSISS3_402 | 59 | hypothetical protein | IIA or IID | 6175 |
|  |  | HSISS3_920 | 73 | hypothetical protein | IID | 6125 |
|  |  | HSISS3_416 | 79 | hypothetical protein | IIA or IID | 6100 |
|  |  | HSISS3_766 | 73 | hypothetical protein | IID | 5925 |
|  |  | HSISS3_769 | 53 | hypothetical protein |  | 1475 |
|  |  | HSISS3_767 | 75 | hypothetical protein | IID | 1275 |
|  |  | HSISS3_921 | 75 | hypothetical protein | IID | 1125 |
|  |  | HSISS3_410 | 51 | hypothetical protein |  | 1100 |
|  |  | HSISS3_923 | 53 | hypothetical protein |  | 1050 |
|  |  | HSISS3_45 | 183 | hypothetical protein |  | 1025 |
|  | 4 | HSISS4_689 | 105 | Cytidine deaminase (EC 3.5.4.5) |  | 1100 |
|  |  | HSISS4_1985 | 98 | Acetyltransferase (EC 2.3.1.-) |  | 1100 |
|  |  | HSISS4_1831 | 76 | Bacteriocin BlpU | IIA | 5100 |
|  |  | HSISS4_1840 | 76 | hypothetical protein | IIA | 5100 |

*: Candidates with ≥1800 points are highlighted in grey and are considered ‘putative bacteriocins’ by BAGEL2 [[1](#_ENREF_1)] while a score below this threshold value but with a score of 1000 are considered ‘interesting candidates’.

REFERENCES

1. de Jong A, van Heel AJ, Kok J, Kuipers OP (2010) BAGEL2: mining for bacteriocins in genomic data. Nucleic Acids Res 38: W647-651.
