# Supplementary material for: Comparative Genomics Analysis of Streptococcus Isolates from the Human Small Intestine Reveals their Adaptation to a Highly Dynamic Ecosystem
Source: PLoS One. 2013 Dec 30;8(12):e83418. doi: 10.1371/journal.pone.0083418 (PMC3875467; doi:10.1371/journal.pone.0083418)
Supplement: Table S11 — Locus tags of enzymes involved in glycolysis and pentose phosphate pathway. (DOCX) [file pone.0083418.s014.docx]

Table S11: Locus tags of enzymes involved in glycolysis and pentose phosphate pathway

| Product | EC number | *S. parasanguinis* | *S. equinus* | *S. salivarius* group | | | |
| --- | --- | --- | --- | --- | --- | --- | --- |
|  |  |  |  | 1 | 2 | 3 | 4 |
| Glucokinase^a^ | 2.7.1.2 | HSISM1_1367 | HSISB1_1465 | HSISS1_1788 | HSISS2_726 | HSISS3_2176 | HSISS4_887 |
| Glucose-6-phosphate isomerase^a,b^ | 5.3.1.9 | HSISM1_281 | HSISB1_1539 | HSISS1_311 | HSISS2_516 | HSISS3_1145 | HSISS4_187 |
| 6-phosphofructokinase 1^a^ | 2.7.1.11 | HSISM1_1443 | HSISB1_971 | HSISS1_1897 | HSISS2_1468 | HSISS3_1331 | HSISS4_762 |
| Fructose-bisphosphate aldolase class II^a^ | 4.1.2.13 | HSISM1_392 | HSISB1_443 | HSISS1_140 | HSISS2_1041 | HSISS3_94 | HSISS4_331 |
| glucose-6-phosphate 1-dehydrogenase^b^ | 1.1.1.49 | HSISM1_1750 |  |  |  |  |  |
| 6-phosphogluconolactonase^b^ | 3.1.1.31 | HSISM1_1690 |  |  |  |  |  |
| 6-phosphogluconate dehydrogenase^b^ | 1.1.1.44 | HSISM1_1918 |  |  |  |  |  |
| ribulose-phosphate 3-epimerase^b^ | 5.1.3.1 | HSISM1_47 | HSISB1_1612  HSISB1_1613 | HSISS1_1603 | HSISS2_2154 | HSISS3_241 | HSISS4_1953 |
| Xylulose-5-phosphate phosphoketolase^b^ | 4.1.2.9 | HSISM1_2010 | HSISB1_150  HSISB1_151 |  |  |  |  |
| glyceraldehyde 3-phosphate dehydrogenase^a,b^ | 1.2.1.12 | HSISM1_37 | HSISB1_1622 | HSISS1_1595 | HSISS2_2144 | HSISS3_292 | HSISS4_1945 |
| phosphoglycerate kinase^a,b^ | 2.7.2.3 | HSISM1_35 | HSISB1_1623  HSISB1_1624 | HSISS1_1593 | HSISS2_2143 | HSISS3_293 | HSISS4_1943 |
| glyceraldehyde-3-phosphate dehydrogenase (NADP) ^a,b^ | 1.2.1.9 |  | HSISB1_1346 | HSISS1_1810 | HSISS2_696  HSISS2_697 | HSISS3_2201  HSISS3_2202 | HSISS4_861  HSISS4_862 |
| 2,3-bisphosphoglycerate-dependent phosphoglycerate mutase^a,b^ | 5.4.2.1 | HSISM1_797 | HSISB1_1415 | HSISS1_1891 | HSISS2_1477 | HSISS3_1323 | HSISS4_769 |
| enolase^a,b^ | 4.2.1.11 | HSISM1_1692 | HSISB1_2143 | HSISS1_1650 | HSISS2_428 | HSISS3_2044 | HSISS4_1033 |
| pyruvate kinase^a,b^ | 2.7.1.40 | HSISM1_1444 | HSISB1_970 | HSISS1_1898 | HSISS2_1467 | HSISS3_1332 | HSISS4_761 |
| Phosphate acetyltransferase^b^ | 2.3.1.8 | HSISM1_1190 | HSISB1_943 | HSISS1_1241 | HSISS2_1771 | HSISS3_656 | HSISS4_1592 |
| Acetaldehyde dehydrogenase^b^ | 1.2.1.10 | HSISM1_707 | HSISB1_260  HSISB1_261  HSISB1_262 | HSISS1_130 | HSISS2_1031  HSISS2_1032 | HSISS3_86  HSISS3_87 | HSISS4_323 |
| Alcohol dehydrogenase^b^ | 1.1.1.1 | HSISM1_2035  HSISM1_592  HSISM1_593  HSISM1_707 | HSISB1_1673  HSISB1_260  HSISB1_261  HSISB1_262 | HSISS1_130  HSISS1_756  HSISS1_819  HSISS1_820 | HSISS2_1031  HSISS2_1032  HSISS2_1122  HSISS2_1123  HSISS2_848 | HSISS3_1570  HSISS3_1621  HSISS3_86  HSISS3_87 | HSISS4_1123  HSISS4_323  HSISS4_478 |

^a^: Enzyme involved in glycolysis

^b^: Enzyme involved in pentose phosphate pathway
