# Supplementary material for: Comparative Genomics Analysis of Streptococcus Isolates from the Human Small Intestine Reveals their Adaptation to a Highly Dynamic Ecosystem
Source: PLoS One. 2013 Dec 30;8(12):e83418. doi: 10.1371/journal.pone.0083418 (PMC3875467; doi:10.1371/journal.pone.0083418)
Supplement: Table S12 — Comparison of isolate groupings from genetic fingerprinting and results from lineage-specific PCRs. (DOCX) [file pone.0083418.s015.docx]

Table S12: Comparison of isolate groupings from genetic fingerprinting and results from lineage-specific PCRs

| Isolate | Genus identification | Grouping according to AFLP and Rep-PCR analysis* | | | | | | Grouping according to strain-specific PCR assays | | | | | |
| --- | --- | --- | --- | --- | --- | --- | --- | --- | --- | --- | --- | --- | --- |
|  |  | *S. parasanguinis* | *S. equinus* | *S. salivarius* | | | | *S. parasanguinis* | *S. equinus* | *S. salivarius* | | | |
|  |  |  |  | 1 | 2 | 3 | 4 |  |  | 1 | 2 | 3 | 4 |
| 2010_Ileo_MS_Ia | *Streptococcus* |  |  |  |  |  |  |  |  |  |  |  |  |
| 2010_Ileo_MS_Ib | *Streptococcus* |  |  |  |  |  |  |  |  |  |  |  |  |
| 2010_Ileo_MS_Ic | *Streptococcus* |  |  |  |  |  |  |  |  |  |  |  |  |
| 2010_Ileo_MS_Id | *Streptococcus* |  |  |  |  |  |  |  |  |  |  |  |  |
| 2010_Ileo_MS_Ie | *Streptococcus* |  |  |  |  |  |  |  |  |  |  |  |  |
| 2010_Ileo_MS_If | *Streptococcus* |  |  |  |  |  |  |  |  |  |  |  |  |
| 2010_Ileo_MS_IIa | *Streptococcus* |  |  |  |  |  |  |  |  |  |  |  |  |
| 2010_Ileo_MS_IIb | *Streptococcus* |  |  |  |  |  |  |  |  |  |  |  |  |
| 2010_Ileo_MS_IIc | *Streptococcus* |  |  |  |  |  |  |  |  |  |  |  |  |
| 2010_Ileo_MS_IId | *Streptococcus* |  |  |  |  |  |  |  |  |  |  |  |  |
| 2010_Ileo_MS_IIe | *Streptococcus* |  |  |  |  |  |  |  |  |  |  |  |  |
| 2010_Ileo_MS_IIf | *Streptococcus* |  |  |  |  |  |  |  |  |  |  |  |  |
| 2010_Ileo_MS_IIIa | *Streptococcus* |  |  |  |  |  |  |  |  |  |  |  |  |
| 2010_Ileo_MS_IIIb | *Streptococcus* |  |  |  |  |  |  |  |  |  |  |  |  |
| 2010_Ileo_MS_IIIc | *Streptococcus* |  |  |  |  |  |  |  |  |  |  |  |  |
| 2010_Ileo_MS_IIId | *Streptococcus* |  |  |  |  |  |  |  |  |  |  |  |  |
| 2010_Ileo_MS_IIIe | *Streptococcus* |  |  |  |  |  |  |  |  |  |  |  |  |
| 2010_Ileo_MS_IIIf | *Streptococcus* |  |  |  |  |  |  |  |  |  |  |  |  |
| 2010_Ileo_MS_IVa | *Enterococcus* |  |  |  |  |  |  |  |  |  |  |  |  |
| 2010_Ileo_MS_IVb | *Enterococcus* |  |  |  |  |  |  |  |  |  |  |  |  |
| 2010_Ileo_MS_IVc | *Enterococcus* |  |  |  |  |  |  |  |  |  |  |  |  |
| 2010_Ileo_MS_IVd | *Enterococcus* |  |  |  |  |  |  |  |  |  |  |  |  |
| 2010_Ileo_MS_IVe | *Enterococcus* |  |  |  |  |  |  |  |  |  |  |  |  |
| 2010_Ileo_MS_IVf | *Enterococcus* |  |  |  |  |  |  |  |  |  |  |  |  |
| 2010_Ileo_MS_Va | *Streptococcus* |  |  |  |  |  |  |  |  |  |  |  |  |
| 2010_Ileo_MS_Vb | *Enterococcus* |  |  |  |  |  |  |  |  |  |  |  |  |
| 2010_Ileo_MS_Vc | *Streptococcus* |  |  |  |  |  |  |  |  |  |  |  |  |
| 2010_Ileo_MS_Vd | *Streptococcus* |  |  |  |  |  |  |  |  |  |  |  |  |
| 2010_Ileo_MS_Ve | *Streptococcus* |  |  |  |  |  |  |  |  |  |  |  |  |
| 2010_Ileo_MS_Vf | *Streptococcus* |  |  |  |  |  |  |  |  |  |  |  |  |
| 2010_Ileo_MS_VIa | *Enterococcus* |  |  |  |  |  |  |  |  |  |  |  |  |
| 2010_Ileo_MS_VIb | *Streptococcus* |  |  |  |  |  |  |  |  |  |  |  |  |
| 2010_Ileo_MS_VIc | *Streptococcus* |  |  |  |  |  |  |  |  |  |  |  |  |
| 2010_Ileo_MS_VId | *Streptococcus* |  |  |  |  |  |  |  |  |  |  |  |  |
| 2010_Ileo_MS_VIe | *Streptococcus* |  |  |  |  |  |  |  |  |  |  |  |  |
| 2010_Ileo_MS_VIf | *Streptococcus* |  |  |  |  |  |  |  |  |  |  |  |  |
| 2010_Ileo_MS_VIIa | *Streptococcus* |  |  |  |  |  |  |  |  |  |  |  |  |
| 2010_Ileo_MS_VIIb | *Streptococcus* |  |  |  |  |  |  |  |  |  |  |  |  |
| 2010_Ileo_MS_VIIc | *Streptococcus* |  |  |  |  |  |  |  |  |  |  |  |  |
| 2010_Ileo_MS_VIId | *Enterococcus* |  |  |  |  |  |  |  |  |  |  |  |  |
| 2010_Ileo_MS_VIIe | *Streptococcus* |  |  |  |  |  |  |  |  |  |  |  |  |
| 2010_Ileo_MS_VIIf | *Streptococcus* |  |  |  |  |  |  |  |  |  |  |  |  |
| 2010_Ileo_MS_VIIIa | *Streptococcus* |  |  |  |  |  |  |  |  |  |  |  |  |
| 2010_Ileo_MS_VIIIb | *Streptococcus* |  |  |  |  |  |  |  |  |  |  |  |  |
| 2010_Ileo_MS_VIIIc | *Streptococcus* |  |  |  |  |  |  |  |  |  |  |  |  |
| 2010_Ileo_MS_VIIId | *Streptococcus* |  |  |  |  |  |  |  |  |  |  |  |  |
| 2010_Ileo_MS_VIIIe | *Streptococcus* |  |  |  |  |  |  |  |  |  |  |  |  |
| 2010_Ileo_MS_VIIIf | *Streptococcus* |  |  |  |  |  |  |  |  |  |  |  |  |
| 2010_Ileo_MS_IXa | *Streptococcus* |  |  |  |  |  |  |  |  |  |  |  |  |
| 2010_Ileo_MS_IXb | *Streptococcus* |  |  |  |  |  |  |  |  |  |  |  |  |
| 2010_Ileo_MS_IXc | *Streptococcus* |  |  |  |  |  |  |  |  |  |  |  |  |
| 2010_Ileo_MS_IXd | *Streptococcus* |  |  |  |  |  |  |  |  |  |  |  |  |
| 2010_Ileo_MS_IXe | *Streptococcus* |  |  |  |  |  |  |  |  |  |  |  |  |
| 2010_Ileo_MS_IXf | *Streptococcus* |  |  |  |  |  |  |  |  |  |  |  |  |
| 2010_Ileo_MS_Xa | *Enterococcus* |  |  |  |  |  |  |  |  |  |  |  |  |
| 2010_Ileo_MS_Xb | *Enterococcus* |  |  |  |  |  |  |  |  |  |  |  |  |
| 2010_Ileo_MS_Xc | *Enterococcus* |  |  |  |  |  |  |  |  |  |  |  |  |
| 2010_Ileo_MS_Xd | *Enterococcus* |  |  |  |  |  |  |  |  |  |  |  |  |
| 2010_Ileo_MS_Xe | *Enterococcus* |  |  |  |  |  |  |  |  |  |  |  |  |
| 2010_Ileo_MS_Xf | *Streptococcus* |  |  |  |  |  |  |  |  |  |  |  |  |
| 2010_Ileo_MS_XIa | *Streptococcus* |  |  |  |  |  |  |  |  |  |  |  |  |
| 2010_Ileo_MS_XIb | *Streptococcus* |  |  |  |  |  |  |  |  |  |  |  |  |
| 2010_Ileo_MS_XIc | *Streptococcus* |  |  |  |  |  |  |  |  |  |  |  |  |
| 2010_Ileo_MS_XId | *Streptococcus* |  |  |  |  |  |  |  |  |  |  |  |  |
| 2010_Ileo_MS_XIe | *Streptococcus* |  |  |  |  |  |  |  |  |  |  |  |  |
| 2010_Ileo_MS_XIf | *Streptococcus* |  |  |  |  |  |  |  |  |  |  |  |  |
| 2010_Ileo_MS_XIIa | *Enterococcus* |  |  |  |  |  |  |  |  |  |  |  |  |
| 2010_Ileo_MS_XIIb | *Enterococcus* |  |  |  |  |  |  |  |  |  |  |  |  |
| 2010_Ileo_MS_XIIc | *Streptococcus* |  |  |  |  |  |  |  |  |  |  |  |  |
| 2010_Ileo_MS_XIId | *Streptococcus* |  |  |  |  |  |  |  |  |  |  |  |  |
| 2010_Ileo_MS_XIIe | *Enterococcus* |  |  |  |  |  |  |  |  |  |  |  |  |
| 2010_Ileo_MS_XIIf | *Enterococcus* |  |  |  |  |  |  |  |  |  |  |  |  |
| 2010_Ileo_MS_XIIIa | *Streptococcus* |  |  |  |  |  |  |  |  |  |  |  |  |
| 2010_Ileo_MS_XIIIb | *Streptococcus* |  |  |  |  |  |  |  |  |  |  |  |  |
| 2010_Ileo_MS_XIIIc | *Streptococcus* |  |  |  |  |  |  |  |  |  |  |  |  |
| 2010_Ileo_MS_XIIId | *Streptococcus* |  |  |  |  |  |  |  |  |  |  |  |  |
| 2010_Ileo_MS_XIIIe | *Streptococcus* |  |  |  |  |  |  |  |  |  |  |  |  |
| 2010_Ileo_MS_XIIIf | *Streptococcus* |  |  |  |  |  |  |  |  |  |  |  |  |
| 2010_Ileo_MS_XIVa | *Enterococcus* |  |  |  |  |  |  |  |  |  |  |  |  |
| 2010_Ileo_MS_XIVb | *Streptococcus* |  |  |  |  |  |  |  |  |  |  |  |  |
| 2010_Ileo_MS_XIVc | *Enterococcus* |  |  |  |  |  |  |  |  |  |  |  |  |
| 2010_Ileo_MS_XIVd | *Enterococcus* |  |  |  |  |  |  |  |  |  |  |  |  |
| 2010_Ileo_MS_XIVe | *Enterococcus* |  |  |  |  |  |  |  |  |  |  |  |  |
| 2010_Ileo_MS_XIVf | *Streptococcus* |  |  |  |  |  |  |  |  |  |  |  |  |
| 2010_Ileo_MS_XVa | *Streptococcus* |  |  |  |  |  |  |  |  |  |  |  |  |
| 2010_Ileo_MS_XVb | *Streptococcus* |  |  |  |  |  |  |  |  |  |  |  |  |
| 2010_Ileo_MS_XVc | *Streptococcus* |  |  |  |  |  |  |  |  |  |  |  |  |
| 2010_Ileo_MS_XVd | *Enterococcus* |  |  |  |  |  |  |  |  |  |  |  |  |
| 2010_Ileo_MS_XVe | *Streptococcus* |  |  |  |  |  |  |  |  |  |  |  |  |
| 2010_Ileo_MS_XVf | *Streptococcus* |  |  |  |  |  |  |  |  |  |  |  |  |
| 2010_Ileo_MS_XVIa | *Streptococcus* |  |  |  |  |  |  |  |  |  |  |  |  |
| 2010_Ileo_MS_XVIb | *Streptococcus* |  |  |  |  |  |  |  |  |  |  |  |  |
| 2010_Ileo_MS_XVIc | *Streptococcus* |  |  |  |  |  |  |  |  |  |  |  |  |
| 2010_Ileo_MS_XVId | *Streptococcus* |  |  |  |  |  |  |  |  |  |  |  |  |
| 2010_Ileo_MS_XVIe | *Streptococcus* |  |  |  |  |  |  |  |  |  |  |  |  |
| 2010_Ileo_MS_XVIf | *Streptococcus* |  |  |  |  |  |  |  |  |  |  |  |  |
| 2010_Ileo_MS_XVIIa | *Streptococcus* |  |  |  |  |  |  |  |  |  |  |  |  |
| 2010_Ileo_MS_XVIIb | *Streptococcus* |  |  |  |  |  |  |  |  |  |  |  |  |
| 2010_Ileo_MS_XVIIc | *Streptococcus* |  |  |  |  |  |  |  |  |  |  |  |  |
| 2010_Ileo_MS_XVIId | *Streptococcus* |  |  |  |  |  |  |  |  |  |  |  |  |
| 2010_Ileo_MS_XVIIe | *Streptococcus* |  |  |  |  |  |  |  |  |  |  |  |  |
| 2010_Ileo_MS_XVIIf | *Streptococcus* |  |  |  |  |  |  |  |  |  |  |  |  |
| 2010_Ileo_MS_XVIIIa | *Streptococcus* |  |  |  |  |  |  |  |  |  |  |  |  |
| 2010_Ileo_MS_XVIIIb | *Enterococcus* |  |  |  |  |  |  |  |  |  |  |  |  |
| 2010_Ileo_MS_XVIIIc | *Enterococcus* |  |  |  |  |  |  |  |  |  |  |  |  |
| 2010_Ileo_MS_XVIIId | *Enterococcus* |  |  |  |  |  |  |  |  |  |  |  |  |
| 2010_Ileo_MS_XVIIIe | *Enterococcus* |  |  |  |  |  |  |  |  |  |  |  |  |
| 2010_Ileo_MS_XVIIIf | *Enterococcus* |  |  |  |  |  |  |  |  |  |  |  |  |
| 2010_Ileo_MS_XIXa | *Streptococcus* |  |  |  |  |  |  |  |  |  |  |  |  |
| 2010_Ileo_MS_XIXb | *Streptococcus* |  |  |  |  |  |  |  |  |  |  |  |  |
| 2010_Ileo_MS_XIXc | *Streptococcus* |  |  |  |  |  |  |  |  |  |  |  |  |
| 2010_Ileo_MS_XIXd | *Streptococcus* |  |  |  |  |  |  |  |  |  |  |  |  |
| 2010_Ileo_MS_XIXe | *Streptococcus* |  |  |  |  |  |  |  |  |  |  |  |  |
| 2010_Ileo_MS_XIXf | *Streptococcus* |  |  |  |  |  |  |  |  |  |  |  |  |
| 2010_Ileo_MS_XXa | *Streptococcus* |  |  |  |  |  |  |  |  |  |  |  |  |
| 2010_Ileo_MS_XXb | *Streptococcus* |  |  |  |  |  |  |  |  |  |  |  |  |
| 2010_Ileo_MS_XXc | *Streptococcus* |  |  |  |  |  |  |  |  |  |  |  |  |
| 2010_Ileo_MS_XXd | *Streptococcus* |  |  |  |  |  |  |  |  |  |  |  |  |
| 2010_Ileo_MS_XXe | *Streptococcus* |  |  |  |  |  |  |  |  |  |  |  |  |
| 2010_Ileo_MS_XXf | *Streptococcus* |  |  |  |  |  |  |  |  |  |  |  |  |
| Total number of isolate per lineage |  | 3 | 13 | 1 | 1 | 8 | 66 | 3 | 13 | 57 | 1 | 8 | 67 |

*: See [[1](#_ENREF_1)]

REFERENCES

1. van den Bogert B, Erkus O, Boekhorst J, de Goffau M, Smid EJ, et al. (2013) Diversity of human small intestinal *Streptococcus* and *Veillonella* populations. FEMS Microbiol Ecol 85: 376-388.
